# Traditional Medicinal Plant Knowledge in the Guba District of Azerbaijan: An Ethnobotanical Study

**DOI:** 10.3390/plants15193009

**Published:** 2026-09-30

**Authors:** Javanshir Isayev, Sabina Aliyeva, Elmir Shabayev, Leyla Mansurova, Naila Sultanova, Gunay Jafarova, Nilufar Safarova

**Affiliations:** 1 Department of Pharmacognosy, Azerbaijan Medical University, Baku AZ1022, Azerbaijan; isayev.cavanshir@amu.edu.az (J.I.); seliyeva5@amu.edu.az (S.A.); nilupharm@gmail.com (N.S.); 2 Center for Data Science, New York University, New York, NY 10011, USA; es5902@nyu.edu; 3 Department of Pharmaceutical Technology & Administration, Azerbaijan Medical University, Baku AZ1022, Azerbaijan; lmansurova@amu.edu.az; 4 Department of Children’s Diseases II, Azerbaijan Medical University, Baku AZ1022, Azerbaijan; nsultanova@amu.edu.az

**Keywords:** ethnobotany, ethnomedicine, traditional medicinal knowledge, medicinal plants, Caucasian flora, Guba District

## Abstract

Medicinal plants remain important components of rural healthcare and everyday household practices in Azerbaijan, yet their uses remain insufficiently documented in many culturally diverse regions. This study investigated which medicinal plants are used in the Guba District, how their uses vary according to informant characteristics and ecological setting, and which species–use records may differ from those documented elsewhere in the Caucasus. During 2024–2025, semi-structured interviews were conducted with 260 informants from 40 villages representing Azerbaijani, Lezgi, Tat, Khinalug, Kryts, Jek, and Mountain Jewish (Juhuri-speaking) communities. The principal dataset comprised 193 medicinal plant taxa belonging to 170 genera and 59 families, based on 7385 informant–taxon citations and 8682 use-reports. Eleven additional taxa, each cited by one to four informants within a single settlement, were documented separately as rare, locality-specific records. Descriptive comparisons showed that traditional healers reported broader medicinal plant repertoires than other community members, whereas differences according to gender, education, and ecological zone were comparatively modest. Comparison with selected primary ethnobotanical studies from Azerbaijan and neighboring Caucasian regions identified 36 species–use records as potentially novel within the defined comparative corpus. Thirty-five medicinal taxa were also used as foods and 22 as melliferous plants, demonstrating that local medicinal knowledge is embedded in dietary practices, beekeeping, animal husbandry, and other household activities. Twenty-four medicinal taxa were included in Azerbaijan’s national conservation lists. These findings demonstrate the biocultural importance of Guba’s medicinal flora and support community-centered multilingual documentation, safety awareness, and sustainable-harvesting initiatives.

## 1. Introduction

Plant-derived preparations have served as principal therapeutic agents in human medicine since ancient times [1,2]. Medicinal plants continue to play a fundamental role in the prevention and treatment of numerous diseases and remain an important component of traditional healthcare systems worldwide, particularly in communities where access to conventional healthcare services may be limited [3,4]. However, this long-established tradition is increasingly threatened by ecological and social change. Unsustainable harvesting, habitat degradation, land-use change, and other anthropogenic pressures are contributing to the depletion of medicinal plant resources, while the knowledge associated with their identification, collection, preparation, and therapeutic use is gradually being lost. The preservation and transmission of this knowledge are influenced by geographical setting, ethnic identity, educational background, socioeconomic conditions, and access to modern healthcare services [5]. In many settings, traditional medicinal knowledge is retained predominantly by older generations and may be affected by weakening intergenerational transmission; consequently, its systematic documentation remains important [6,7].

Documenting the medicinal use of plants by local communities is important not only for preserving biocultural heritage but also for providing a scientific basis for future phytochemical, pharmacological, and pharmaceutical research [2,8]. Quantitative ethnobotanical approaches further facilitate the identification of culturally important medicinal plant species, enable assessment of their relative importance within local communities, and reveal their principal therapeutic applications. Such information can support the prioritization of promising taxa for targeted phytochemical and pharmacological investigation.

The Caucasus is internationally recognized as a global biodiversity hotspot [9,10] and has a long-standing tradition of medicinal and wild plant use [11,12,13]. Reflecting the region’s substantial biological and cultural diversity, ethnopharmacological research in the Caucasus has expanded considerably. Field investigations have documented the traditional use of medicinal and wild food plants in Georgia [11,12] and Iranian Azerbaijan [13]. Within the Republic of Azerbaijan, studies have examined the influence of ethnic and religious diversity on wild plant use [14] and the preservation of traditional knowledge of wild food plants among mountain communities [15]. Nevertheless, broad ethnopharmacological investigations within the Republic of Azerbaijan remain limited [16,17]. This limited systematic documentation is particularly important given the country’s botanical diversity and multicultural heritage and highlights the need for further ethnopharmacological research across its diverse ecological and cultural regions.

Despite its relatively small territory of 86,600 km^2^, Azerbaijan harbours approximately 4500 plant species [18]. This botanical richness is shaped by the country’s complex topography, diverse climatic conditions, and pronounced altitudinal gradients, which extend from semi-arid lowlands to permanently snow-covered mountain peaks [18]. The northern and northeastern mountainous regions are characterized by broad-leaved forests and alpine and subalpine meadows and are inhabited by several ethnolinguistic communities that have preserved distinct traditions of medicinal plant use. The close association between botanical diversity and cultural heritage is particularly evident in northeastern Azerbaijan, where varied ecological conditions and long-standing traditional knowledge provide a suitable setting for ethnopharmacological research.

The Guba District, situated in northeastern Azerbaijan on the southern slopes of the Greater Caucasus, is characterized by substantial ecological and cultural diversity. The district encompasses lowland, foothill, mid-mountain, and high-mountain landscapes, with elevations ranging from approximately 200 m to more than 4000 m above sea level. A substantial part of the district lies within Shahdag National Park, contributing to the conservation of its plant diversity. Guba also has a multicultural population comprising an Azerbaijani majority alongside Lezgins, Tats, Khinalugs, Kryts, Jeks, and Mountain Jews. Although these communities differ in their cultural backgrounds and historical traditions, Azerbaijani serves as a common language of communication. Several minority languages represented in the study face documented language endangerment. Budukh, Kryz, and Khinalug have been described as endangered to varying degrees, while Juhuri (Judeo-Tat) is likewise described as an endangered minority language [19,20]. The Jek (Cek) community is part of the Kryz-speaking cluster documented in northeastern Azerbaijan [19]. The coexistence of diverse ecological conditions and cultural communities has contributed to the preservation of extensive traditional knowledge concerning the medicinal use of wild plants.

Despite its ecological and cultural significance, comprehensive quantitative ethnopharmacological investigation of the Guba District remains limited. Previous studies in Azerbaijan have focused mainly on particular regions or disease categories, including digestive disorders in the Nakhchivan Highlands [21], respiratory disorders in the Zuvand territory [22], cardiovascular disorders in the upland areas of Guba [23], and urological disorders in the Gadabay region [24]. Published ethnobotanical and wild-plant-use studies from Azerbaijan, Georgia, Dagestan, and Armenia provide a broader comparative context for plant use across the Caucasus [11,12,14,15,25,26,27,28,29,30]. A related comparative study by Pieroni et al. [31] documented wild food plants among seven ethnolinguistic groups in 14 villages in northeastern Georgia and compared findings for Azeri and Udi communities with data collected in Azerbaijan, highlighting the role of linguistic diversity in regional plant-use knowledge. However, to our knowledge, a district-wide quantitative ethnopharmacological assessment encompassing a broad range of settlements, ecological zones, and ethnolinguistic communities has not previously been undertaken. Moreover, much of the region’s ethnomedicinal knowledge is retained by older community members and is not being transmitted to younger generations at the same rate, increasing the risk that this biocultural heritage may disappear before it is adequately documented.

Against this background, the present study aimed to document and analyse medicinal plant knowledge across the culturally and ecologically diverse Guba District. The study addressed the following research questions: (1) Which medicinal plant taxa, botanical families, preparation methods, and therapeutic applications are most prominent within the surveyed ethnomedicinal system? (2) Do recognized traditional healers report a broader range of medicinal plant taxa and use-reports than other community members? (3) How do the numbers of taxa and use-reports reported per informant vary according to gender, educational level, settlement, and ecological zone, and is there an observable pattern along the altitudinal gradient? (4) Which recorded plant uses are shared with or differ from those documented in previous field-based ethnobotanical studies in Azerbaijan and neighbouring Caucasian regions, particularly among Lezgi communities across the Azerbaijan–Dagestan border? (5) Which documented medicinal taxa are of particular conservation concern or represent potentially novel ethnomedicinal reports requiring further investigation? Because the ethnolinguistic groups were represented by markedly unequal sample sizes, formal statistical comparisons of their medicinal plant repertoires were not undertaken; instead, ethnolinguistic and locality-specific knowledge was evaluated through vernacular names, geographically restricted reports, and comparison with previous regional studies.

## 2. Results

### 2.1. Demographic Profile of Informants

**Figure 1:**
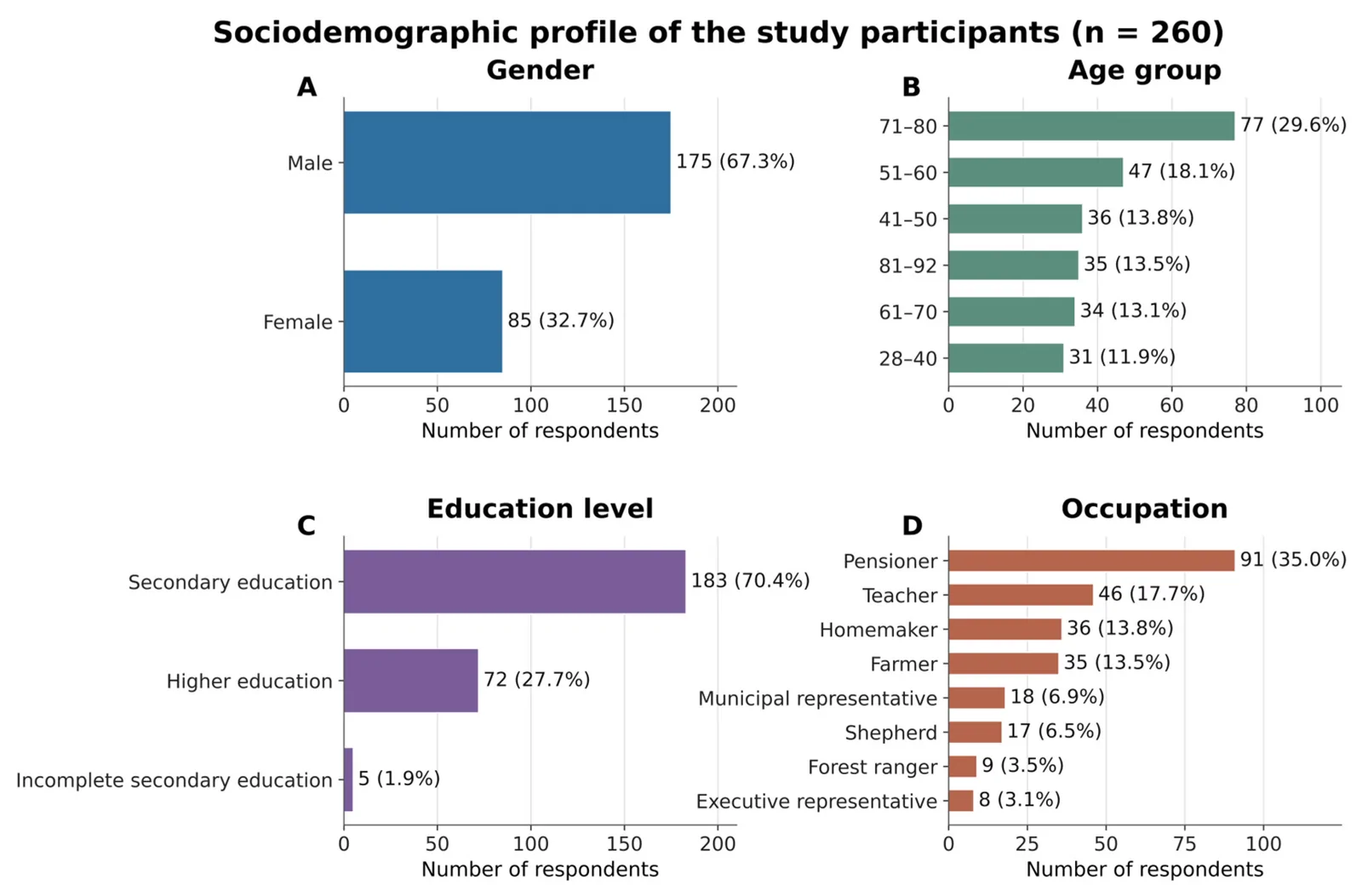
Sociodemographic profile of the study participants (*n* = 260): (**A**) gender distribution; (**B**) age-group distribution; (**C**) education-level distribution; and (**D**) occupational distribution. Values represent the number and percentage of informants in each category.

A total of 260 informants from 40 villages in the Guba District participated in the study. Their demographic characteristics are summarized in Table 1 and Figure 1. Participants ranged in age from 28 to 92 years and represented the major ethnic communities of the district, including Azerbaijanis (*n* = 167; 64.2%), Lezgins (*n* = 23; 8.8%), Tats (*n* = 20; 7.7%), Khinalugs (*n* = 19; 7.3%), Mountain Jews (*n* = 15; 5.8%), Kryts (*n* = 9; 3.5%), and Jeks (*n* = 7; 2.7%). Among the respondents, 17 were identified as key informants based on their recognized expertise in the traditional use of medicinal plants. Most were well-known traditional healers within their respective communities and ranged in age from 61 to 92 years.

Ethnobotanical knowledge was further examined descriptively according to healer status, gender, educational level, and ecological zone (Table 2). Traditional healers were analyzed separately from other community members to avoid obscuring differences associated with specialized ethnomedicinal experience.

Traditional healers generally reported broader ethnomedicinal knowledge than other community members. The 17 traditional healers reported a median of 36 medicinal plant taxa and 41 use-reports per informant, compared with 28 taxa and 32 use-reports among the 243 other community members. Substantial variation was nevertheless observed within the healer group.

Men reported slightly higher median numbers of taxa and use-reports than women, although the difference was modest and should be considered in relation to the unequal gender composition of the sample. Differences according to educational level were also limited. Informants with secondary education reported a median of 28 taxa and 34 use-reports, whereas those with higher education reported 26.5 taxa and 30.5 use-reports. The incomplete-secondary education group included only five informants and was therefore too small to support a meaningful comparison.

Median values were broadly similar across ecological zones, and no clear directional pattern was evident along the altitudinal gradient. Although the highest medians were observed in the foothill and low-mountain zone, this category included only seven informants. Overall, healer status showed a more pronounced descriptive difference than gender, educational level, or ecological zone within the surveyed sample. Descriptive variation was also observed across the self-identified ethnolinguistic groups (Table 2). Tat and Lezgi informants showed the highest median numbers of reported medicinal plant species (32 for both groups) and use-reports (36 for both groups). The corresponding medians were 28 species and 33 use-reports among Azerbaijani informants, 28 and 32 among Khinalug informants, 27 and 31 among Mountain Jewish informants, 23 and 27 among Kryts informants, and 25 and 31 among Jek informants. These values describe the surveyed participants only and were not subjected to inferential comparison because of unequal group sizes and the limited numbers of informants in several minority categories.

### 2.2. Taxonomic Diversity of Medicinal Plants

A total of 193 medicinal plant species belonging to 170 genera and 59 families were documented in the traditional medicine of the Guba District. Their family-level distribution is summarized in Table 3, whereas detailed species-level ethnobotanical data are presented in Table 4. The five most species-rich families were Lamiaceae (22 species), Asteraceae (19 species), Rosaceae (18 species), Fabaceae (13 species), and Apiaceae (10 species), together representing 42.5% of all recorded species. Twenty-four families were represented by a single species each: Amaranthaceae, Asphodelaceae, Berberidaceae, Cannabaceae, Capparaceae, Cornaceae, Cupressaceae, Equisetaceae, Juglandaceae, Linaceae, Lythraceae, Nitrariaceae, Onagraceae, Pedaliaceae, Portulacaceae, Primulaceae, Rutaceae, Salicaceae, Santalaceae, Sapindaceae, Taxaceae, Thymelaeaceae, Verbenaceae, and Urticaceae. A total of 8682 medicinal use-reports were recorded for the 193 species included in the principal quantitative dataset. The sum of the species-level use-report values presented in Table 4 corresponded exactly to the family-level total presented in Table 3.

The total number of use-reports varied considerably among plant families, ranging from 13 for Santalaceae to 1303 for Lamiaceae (Table 3).

#### Rare and Locality-Specific Ethnomedicinal Reports

In addition to the 193 medicinal plant species retained in the principal quantitative dataset, 11 taxa were reported by only one to four informants within a single settlement. Because these reports were highly localized and based on a small number of citations, they were not included in the principal index-based comparisons. Nevertheless, they are presented here because they may represent culturally and geographically specific ethnomedicinal knowledge (Table 5).

Although these taxa were not included in the principal index-based analyses, their restricted geographical distribution and low citation frequencies may reflect localized or declining knowledge. Their documentation is therefore important for preserving community-specific ethnomedicinal information and for guiding future targeted field investigations.

### 2.3. Life Forms of Medicinal Plants

Herbaceous species predominated, accounting for 153 species (79.3%), followed by trees (22 species; 11.4%), shrubs (17 species; 8.8%), and lianas (1 species; 0.5%).

### 2.4. Plant Parts Used

**Figure 2:**
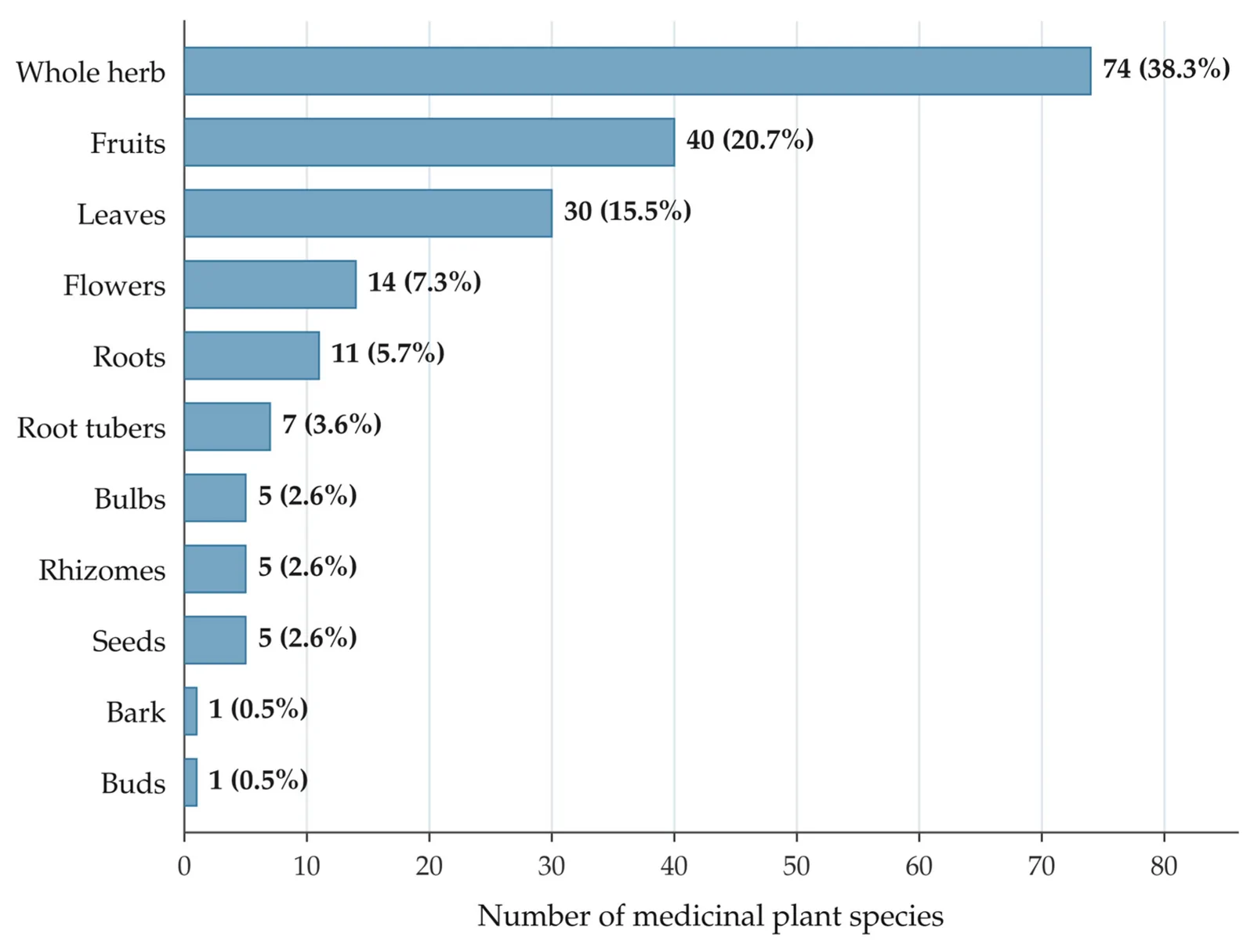
Plant parts used in the traditional medicinal practices of the Guba District (*N* = 193 species).

Eleven categories of plant parts were recorded in the traditional medicinal practices of the Guba District (Figure 2). Aerial parts were used most frequently, with the whole herb (74 species; 38.3%), fruits (40 species; 20.7%), leaves (30 species; 15.5%), and flowers (14 species; 7.3%) together accounting for 81.9% of the documented species. Underground plant parts were used less frequently and included roots (11 species; 5.7%), root tubers (7 species; 3.6%), bulbs (5 species; 2.6%), and rhizomes (5 species; 2.6%). Seeds (5 species; 2.6%), bark (1 species; 0.5%), and buds (1 species; 0.5%) were the least frequently utilized plant parts.

### 2.5. Preparation Methods

**Figure 3:**
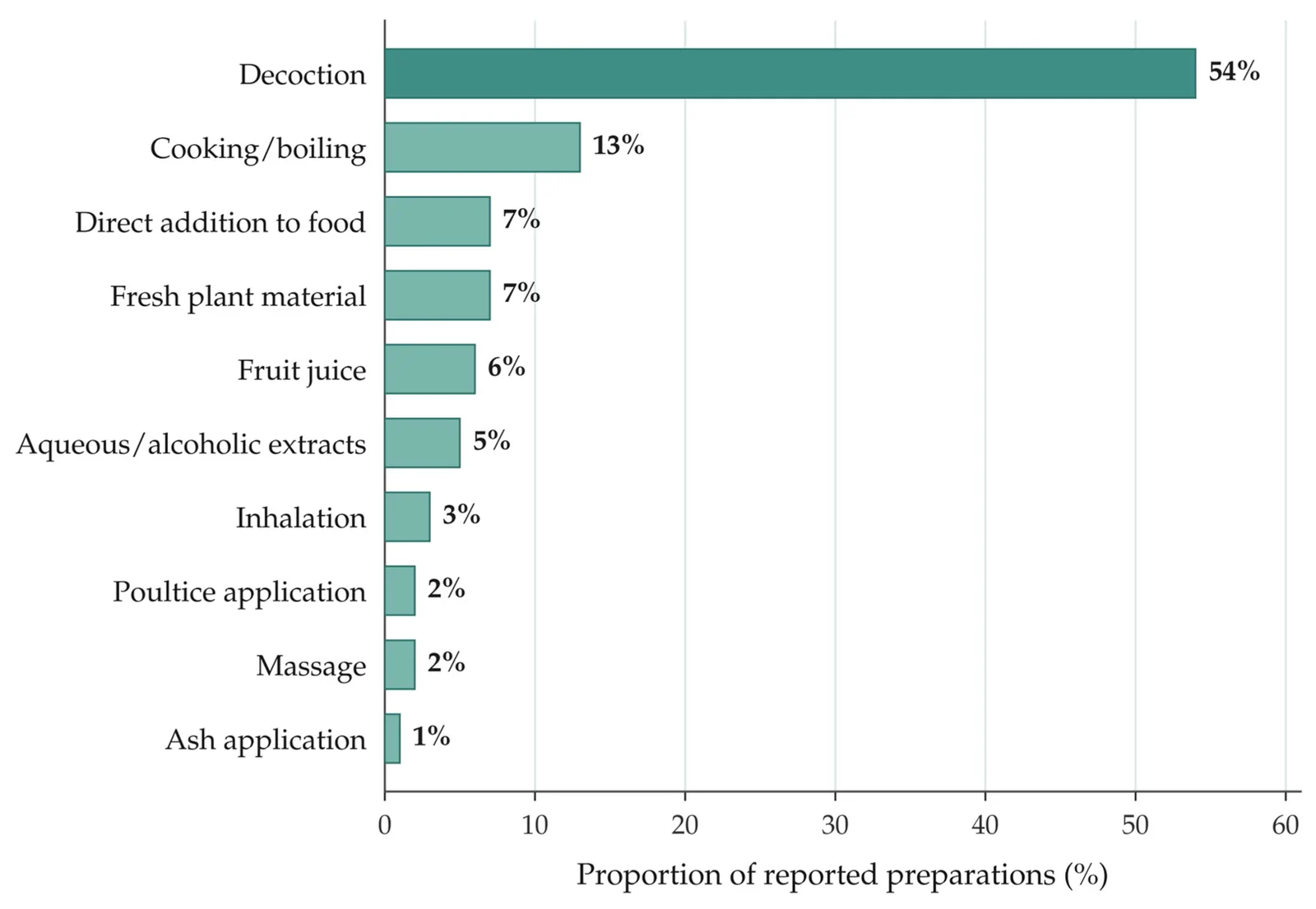
Distribution of preparation methods used in the traditional medicinal practices of the Guba District.

Medicinal plants were prepared using ten different methods (Figure 3). Decoction was the most frequently reported preparation method, accounting for 54% of all reported preparations. Cooking or boiling represented 13%, followed by direct addition to food (7%) and the use of fresh plant material (7%). Fruit juice preparations accounted for 6%, whereas aqueous and alcoholic extracts together represented 5% of all preparations. Less frequently reported methods included inhalation (3%), poultice application (2%), massage (2%), and ash application (1%).

### 2.6. Frequency of Citation, Relative Frequency of Citation, and Use Value

Frequency of Citation (FC), Relative Frequency of Citation (RFC), and Use Value (UV) were calculated for all 193 documented medicinal plant species. FC values ranged from 8 to 97. The highest FC values were recorded for *Rosa tomentosa* Sm. and *Mentha aquatica* L. (97 each), followed by *Glycyrrhiza glabra* L. (84), *Inula helenium* L. (81), *Hippophae rhamnoides* L. (79), *Origanum vulgare* L. (78), and *Anethum graveolens* L. (77).

RFC values ranged from 0.03 to 0.37. The highest RFC values were recorded for *Rosa tomentosa* (0.37), *Mentha aquatica* (0.37), *Glycyrrhiza glabra* (0.32), *Inula helenium* (0.31), and *Hippophae rhamnoides*, *Origanum vulgare*, and *Anethum graveolens* (0.30 each).

UV values ranged from 0.03 to 0.54. The highest UV values were recorded for *Rosa tomentosa* (0.54), *Mentha aquatica* (0.52), *Origanum vulgare* (0.49), *Hippophae rhamnoides* (0.48), *Glycyrrhiza glabra* (0.45), *Inula helenium* (0.39), and *Anethum graveolens* (0.38). The lowest values for all three indices (FC = 8, RFC = 0.03, and UV = 0.03) were recorded for *Campanula sarmatica* Ker-Gawl., *Vicia lathyroides* L., and *Cotoneaster meyeri* Pojark., while Jubogalium setaceum (Lam.) Kadereit & Schneew. and *Scrophularia versicolor* Boiss. each had FC values of 9, with the same RFC (0.03) and UV (0.03).

### 2.7. Fidelity Level

Fidelity Level (FL) values, based on the most frequently reported ailment for each species, ranged from 30.56% to 100%. A total of 84 species were reported for a single ailment category (FL = 100%), including *Allium oreophilum* C.A.Mey., *Allium ursinum* L., *Galanthus alpinus* Sosn. var. *alpinus*, and *Cotinus coggygria* Scop. Among species reported for two or more ailment categories, the highest FL values were recorded for *Valeriana alliariifolia* var. *tiliifolia* (Troickij) V.E.Avet. (93.55%), *Veronica arvensis* L. (93.10%), *Glaucium elegans* Fisch. & C.A.Mey. (93.02%), *Ononis arvensis* L. (92.00%), *Viola caucasica* Kolenati. (90.00%), *Allium sativum* L. (88.68%), and *Prunus dulcis* (Mill.) D.A.Webb (86.79%). The lowest maximum FL values were recorded for *Salvia glutinosa* L. (30.56%), *Satureja hortensis* L. (34.33%), *Melissa officinalis* L. (40.63%), *Alcea kusjariensis* (Iljin ex Grossh.) Iljin (40.91%), and *Hypericum perforatum* L. (41.89%).

### 2.8. Disease Categories and Informant Consensus Factor

Reported medicinal uses were organized into ten broad health-condition categories after the original emic descriptions had been retained in the principal taxa table. The greatest numbers of use-reports were recorded for respiratory system conditions (2057 use-reports), digestive system conditions (1422), genitourinary system conditions (1138), dermatological conditions (1123), and cardiovascular system conditions (855). ICF values across the ten broad categories ranged from 0.945 to 0.971. The highest values were observed for hepatobiliary conditions (ICF = 0.971), nervous system and sleep-related conditions (ICF = 0.970), and respiratory system conditions (ICF = 0.969), whereas the lowest value was recorded for musculoskeletal conditions (ICF = 0.945). The relatively narrow range reflects the large numbers of use-reports in relation to the numbers of taxa within each broad category. These values are therefore presented as descriptive measures of agreement within the surveyed sample and not as evidence of clinical effectiveness or population-wide consensus (Table 6).

### 2.9. Family Use Value

Family Use Value (FUV) was calculated for the 59 documented plant families (Table 4). FUV values ranged from 0.05 to 0.31. The highest FUV values were recorded for Elaeagnaceae (0.31), Hypericaceae and Sapindaceae (0.30 each), and Equisetaceae, Lythraceae, and Moraceae (0.29 each). Among the most species-rich families, FUV values were 0.23 for Lamiaceae, 0.21 for Asteraceae, 0.20 for Rosaceae, 0.18 for Apiaceae, and 0.13 for Fabaceae. The lowest FUV values were recorded for Santalaceae (0.05), Colchicaceae and Oleaceae (0.06 each), and Cupressaceae, Linaceae, Solanaceae, and Thymelaeaceae (0.07 each).

### 2.10. Additional Ethnobotanical Uses

In addition to their medicinal applications, the documented plants were assigned to eight additional ethnobotanical use categories (Table 7). Food use represented the largest category, with 35 species, followed by melliferous use with 22 species. Individual species could occur in more than one category; therefore, the category totals were not additive. Detailed species-level information is provided in Table S2. Among the natural dye plants, specific colors were recorded for several taxa: the fruits of *Rhus coriaria* produced a crimson dye, the fruits of *Sambucus nigra* a dark-purple dye, the flowers of *Calendula arvensis* a dark-orange dye, and the fruit peels of *Punica granatum* a dark-brown dye.

### 2.11. Conservation Status

Among the 193 documented medicinal plant species, 14 species (7.3%) are included in the Red Book of the Republic of Azerbaijan, whereas 10 species (5.2%) are listed in the Pink List of the Republic of Azerbaijan [32] (Table 8). Together, these 24 species represent 12.4% of the documented medicinal flora.

### 2.12. Comparison with Previous Ethnobotanical Studies and Potentially Novel Reports

To assess the regional continuity and potential novelty of the recorded ethnomedicinal knowledge, the species–use combinations documented in the present study were compared with selected primary field-based ethnobotanical studies from Azerbaijan and neighboring regions of the Caucasus, including Dagestan, Georgia, and Armenia. Comparisons were made at the level of the reported medicinal use rather than solely on the presence of a plant species in the literature. Reports not identified in the consulted primary sources were classified cautiously as potentially novel or previously undocumented within the geographical scope of the comparison, rather than as definitively new ethnomedicinal uses (Table 9).

## 3. Discussion

The present study documented 193 medicinal plant species belonging to 59 families in the Guba District, demonstrating the considerable diversity of plant resources incorporated into local traditional healthcare practices. This diversity exceeds the 121 medicinal plant taxa from 44 families recently documented through interviews with 105 informants in the Zagatala District of northwestern Azerbaijan [33] and approaches the 205 species from 60 families reported from the Diabar region of southern Azerbaijan [34]. Direct comparison, however, requires caution because these studies differed in geographical coverage, ecological conditions, numbers of surveyed settlements, and sampling design. In Guba, Lamiaceae, Asteraceae, Rosaceae, Fabaceae, and Apiaceae together accounted for 42.5% of the documented medicinal flora. A recent ethnobotanical survey conducted in the Guba region likewise highlighted Lamiaceae, Rosaceae, and Apiaceae among the principal families represented in local plant use [25]. Although that study was based on a smaller group of expert informants and considered a broader spectrum of useful-plant applications beyond medicinal use, the recurrence of these families in both surveys supports their prominent role in the regional ethnobotanical repertoire. Asteraceae and Lamiaceae were likewise the most species-rich medicinal plant families in Diabar [34], while Rosaceae, Apiaceae, and Lamiaceae were among the leading families reported in neighbouring Dagestan [26]. Their recurrent representation in studies from different parts of Azerbaijan and the wider Caucasus points to a regional pattern, although the relative importance of individual families varies with local flora and cultural practices.

The comparison at the species–use level identified 36 medicinal uses that were not found in the selected ethnobotanical sources consulted for Azerbaijan and neighboring Caucasian regions (Table 9). These records should be regarded as potentially novel within the defined comparative corpus rather than as definitive first reports for the entire Caucasus. Their occurrence may reflect highly localized experimentation, ecological availability, and the transmission of knowledge within particular settlements and family networks. At the same time, the broader overlap of medicinal and food plants reported in Guba and neighboring Caucasian regions suggests a shared regional foundation of plant knowledge, upon which community-specific practices have developed [11,12,14,15,26,27,28,29,30,33,34]. The coexistence of Azerbaijani, Lezgi, Tat, Shahdag, and Mountain Jewish communities may also have facilitated the circulation and reinterpretation of plant-use knowledge; however, the present sampling design does not permit individual practices to be attributed conclusively to a particular ethnolinguistic tradition.

The descriptive comparison according to informant characteristics showed that healer status was associated with the clearest difference in the breadth of reported ethnomedicinal knowledge. Traditional healers reported higher median numbers of both medicinal plant taxa and use-reports than other community members, reflecting their specialized experience and recognized role in preserving and transmitting medicinal plant knowledge. In contrast, the differences observed according to gender and educational level were comparatively modest. Knowledge measures were also broadly similar across ecological zones, with no clear directional pattern along the altitudinal gradient. These findings should be interpreted as patterns within the surveyed sample rather than population-level differences because the purposive and snowball sampling strategy resulted in unequal group sizes.

Ethnolinguistic affiliation likewise showed descriptive variation in the breadth of reported knowledge. Tat and Lezgi informants had the highest median numbers of reported taxa and use-reports, whereas lower medians were observed among the smaller Kryts and Jek groups. These patterns should not be interpreted as evidence that one community possesses inherently greater or more eroded knowledge than another. Group size, age structure, occupation, settlement ecology, accessibility, and the distribution of traditional healers may all influence the observed values. Nevertheless, presenting these groups separately makes visible the contribution of the minority communities to the district’s ethnomedicinal knowledge and provides a basis for future community-specific research. The multilingual vernacular names included in the main taxa table further preserve part of this ethnolinguistic dimension. The documented medicinal flora consisted almost entirely of seed plants. Pteridophytes were represented only by *Equisetum arvense* L. (Equisetaceae; 0.52%), whereas the remaining 192 species (99.48%) were spermatophytes. Of these, two species were gymnosperms and 190 were angiosperms, comprising 11 monocots and 179 eudicots. The predominance of angiosperms was accompanied by a strong representation of herbaceous life forms: herbs accounted for 153 species (79.3%), compared with 22 trees (11.4%), 17 shrubs (8.8%), and one liana (0.5%). Herbaceous dominance has also been observed in other ethnobotanical surveys and may partly reflect the local availability and accessibility of these plants, although growth-form patterns vary with agroecological, topographic, and cultural conditions [35].

Plant-part selection showed a clear preference for aerial materials. Whole-herb material was used for 74 species (38.3%), followed by fruits (40; 20.7%), leaves (30; 15.5%), and flowers (14; 7.3%); together, these materials accounted for 81.9% of the documented species. Underground organs were used considerably less often. A similar emphasis on leaves, aerial parts, flowers, and fruits has been reported from communities on both sides of the Georgia–Turkey border in the Western Lesser Caucasus [27]. Preparation in Guba was also relatively simple, with decoction accounting for 54% of the recorded preparations. Water-based infusions and decoctions are likewise prominent in the Western Lesser Caucasus [27]. Such practices are consistent with a household-based tradition in which medicinal remedies can be prepared using locally available plants and relatively simple processing techniques.

The quantitative indices highlighted a group of species that occupy a particularly prominent place in local medicinal knowledge. High FC and RFC values indicate that a species was cited by a relatively large proportion of informants, whereas a high UV reflects a greater number of reported uses relative to the surveyed population. *Rosa tomentosa* and *Mentha aquatica* had the highest citation frequencies (FC = 97; RFC = 0.37 for both). *Rosa tomentosa* had the highest UV (0.54), followed by *M. aquatica* (0.52). *Origanum vulgare*, *Hippophae rhamnoides*, *Glycyrrhiza glabra*, *Inula helenium*, and *Anethum graveolens* also ranked highly. Its high citation frequency and Use Value in the present study therefore provide quantitative support for its prominence within the ethnomedicinal knowledge of the region, although differences in sampling design preclude direct comparison of citation frequencies between the two studies. The prominence of *R. tomentosa* is noteworthy because the species was also among the most frequently cited medicinal plants in Zagatala (RFC = 0.96) [33]. Likewise, *O. vulgare* has been reported among culturally salient medicinal plants in the Western Lesser Caucasus [27]. These parallels suggest that some components of medicinal plant knowledge are shared across different communities of the Caucasus, even though their relative cultural importance may differ locally. FL adds another dimension by indicating how strongly the reported uses of a species are concentrated on a particular therapeutic purpose. In Guba, 84 species had an FL of 100% because each was recorded for a single reported indication. Thus, high FL in this context reflects use specificity rather than demonstrated therapeutic efficacy. Taken together, FC, RFC, UV, and FL help distinguish widely known, frequently used, and use-specific plants and can guide the selection of culturally important taxa for further investigation.

At the family level, FUV provides information distinct from simple species richness because it reflects the mean Use Value of the species within a family [36]. The families containing the greatest numbers of medicinal species were not necessarily those with the highest FUV. Elaeagnaceae, for example, had the highest FUV (0.31) despite being relatively species-poor, a pattern partly influenced by the high UV of *Hippophae rhamnoides*. This illustrates why taxonomic diversity and ethnobotanical prominence should be considered separately: a family represented by only a few species may still have considerable local importance when those species are frequently or broadly used.

Medicinal knowledge was particularly concentrated around respiratory, digestive, genitourinary, dermatological, and cardiovascular conditions. Respiratory conditions alone accounted for 2057 use-reports, while substantial numbers were also recorded for the other major categories. These figures describe the distribution of ethnomedicinal knowledge and should not be taken as estimates of disease prevalence in the population. The pattern nevertheless fits with earlier ethnopharmacological work in Azerbaijan documenting plant-based treatments for respiratory disorders in Zuvand, digestive disorders in the Nakhchivan Highlands, cardiovascular conditions in the Guba mountain region, and urological disorders in Gadabay [21,22,23,24]. Following reorganization into ten broad, non-overlapping health-condition categories, ICF values ranged from 0.945 to 0.971 and showed limited variation. This narrow range is largely attributable to the high numbers of use-reports relative to the numbers of taxa within the categories. The ICF values should therefore be interpreted as descriptive measures of citation concentration within the surveyed sample rather than as evidence of clinical effectiveness or population-wide agreement. Moreover, broad analytical categories inevitably reduce some of the cultural and semantic detail contained in participants’ accounts; for this reason, the original emic use descriptions have been retained in the principal taxa table. For several plants recorded in Guba, the ethnomedicinal data can be considered alongside existing phytochemical and pharmacological evidence. *Glycyrrhiza glabra*, which had a high UV and was frequently cited for cough and inflammatory conditions, contains bioactive constituents such as glycyrrhizin, isoliquiritigenin, glabridin, and licochalcone A. Experimental pharmacological studies on licorice constituents have demonstrated antitussive activity, providing a pharmacological context for the recorded traditional use against cough [37]. *Hippophae rhamnoides*, another highly ranked species in Guba, was reported for wound healing and dermatological conditions. In vivo experiments have shown that topical formulations containing its fruit oil can enhance wound healing, lending experimental support to this aspect of its traditional use [38]. The medicinal use of *Alchemilla caucasica* recorded in Guba also accords with recent documentation of diverse traditional uses of *Alchemilla* species across several geobotanical regions of Azerbaijan [39].

Earlier phytochemical work on *Ferula persica*, a congener of *F. caspica* recorded in the present survey, identified phenolic derivatives from its roots [40]. Although this genus-level evidence does not directly validate the traditional use of *F. caspica*, it supports the phytochemical potential of *Ferula* taxa and the need for targeted investigation of the locally recorded species.

Previous phytochemical studies on taxa represented in, or closely related to those recorded in, the Guba dataset provide a basis for future targeted investigation but should not be interpreted as validation of the traditional uses documented here. Phenolic derivatives have been reported from *Ferula persica*, a congener of the locally recorded *F. caspica* [40]. Phytochemical profiling and selected biological activities have also been reported for *Gentiana cruciata* and *G. septemfida* [41], while the essential oil of *Dracocephalum botryoides* has been characterized for antimicrobial, antibiofilm, antioxidant, and cytotoxicity-related effects [42]. Because the experimental endpoints examined in these studies do not necessarily correspond to the specific indications reported by Guba informants, these findings are cited as phytochemical context and as a rationale for future use-specific pharmacological studies rather than as evidence validating the recorded traditional uses.

### 3.1. Environmental and Conservation Implications

Conservation is particularly relevant because 24 of the 193 medicinal species (12.4%) are included in the national conservation lists of Azerbaijan, comprising 14 species recorded in the Red Book and 10 in the Pink List of the Republic of Azerbaijan [32]. The presence of traditionally used species among conservation-priority taxa means that cultural importance and biodiversity protection cannot be considered independently. Although underground organs represented a relatively small proportion of the plant materials used, wild collection may still create pressure on particular taxa, especially species with restricted distributions or small populations. More broadly, increasing demand for medicinal plants may contribute to pressure on wild plant resources, highlighting the importance of sustainable sourcing and biodiversity conservation [43]. The issue also extends beyond the plants themselves. Loss of local populations can contribute to the disappearance of the knowledge associated with their recognition, collection, and use. Similar concerns have been raised in the Greater Caucasus, where demographic and socioeconomic changes have been associated with declining traditional ecological knowledge [15]. Sustainable collection, continued documentation, and monitoring of conservation-priority species are therefore important for preserving both the medicinal flora and its associated biocultural heritage.

Conservation concern is not uniform across the 24 nationally listed taxa because the potential impact of harvesting depends strongly on the plant part removed. The collection of underground organs may present the greatest potential risk, particularly for *Ferula caspica* (root), *Ophrys apifera* and *Orchis mascula* (tubers), *Allium ursinum* and *Galanthus alpinus* Sosn. var. *alpinus* (bulbs), and *Colchicum speciosum* (tuber), because removal of these organs may kill the individual plant or impair regeneration. Bark collection from *Quercus castaneifolia* may also cause direct damage when substantial portions are removed. By contrast, the reported collection of fruits from *Punica granatum*, *Rhus coriaria*, and *Sorbus aucuparia*, flowers from *Tanacetum coccineum*, and leaves or aerial parts from the remaining listed taxa may be less destructive when performed selectively, although repeated or intensive harvesting could still affect reproduction and population persistence. Particular caution is also required for the toxic listed taxa *Taxus baccata*, *Atropa bella-donna*, and *Colchicum speciosum*. The survey did not systematically distinguish wild-collected from cultivated material for every species and did not quantify harvesting frequency, traded quantities, or market demand; therefore, the present data do not demonstrate current population-level harvesting pressure. These taxa, especially those harvested for underground organs or bark, should be prioritized for field-based population monitoring and sustainable-harvesting assessment.

### 3.2. Food Sovereignty and the Food–Medicine Continuum

Medicinal plants in Guba also formed part of a wider system of traditional resource use. Thirty-five species were used as food and 22 were recognized as melliferous plants, while other taxa served as spices, fodder, dyes, fuel, construction materials, or ornamentals. This overlap shows that medicinal knowledge is intertwined with food practices, animal husbandry, beekeeping, and household activities rather than functioning as an isolated body of knowledge. This multifunctional character of plant use is consistent with recent field-based research in Azerbaijan, where wild plants were documented as having overlapping food, medicinal, and technical uses [25]. A comparable relationship between local plant diversity and food traditions has been documented in the Greater Caucasus of Azerbaijan, where Sõukand and Pieroni [15] recorded 72 wild food taxa among seven autochthonous linguistic communities. Broadly multifunctional plant use has also been documented in mountain communities of Svaneti and Racha-Lechkhumi in Georgia [11]. The close relationship between wild food-plant knowledge and linguistic and cultural diversity has also been documented among minority communities in northeastern Georgia, further demonstrating the biocultural significance of traditional plant knowledge across the Caucasus [31].

In Guba, informants associated a decline in the traditional use of woody plants as fuel with regional gasification and the establishment of protected areas. This observation illustrates how infrastructure, socioeconomic change, and conservation measures can reshape plant-use practices over time. The overlap between food and medicine was also evident in the preparation data: direct addition to food and the use of fresh plant material each accounted for 7% of the recorded preparations. Together with the 35 species explicitly reported as foods, these practices indicate that part of the local pharmacopoeia operates through everyday dietary routines rather than through remedies prepared only during illness. Wild and locally available plants may therefore contribute simultaneously to household nutrition, self-care, seasonal dietary diversity, and the maintenance of community knowledge. In this context, the food–medicine continuum is relevant to local food sovereignty because it reflects the capacity of households to use culturally familiar biological resources within their own food and healthcare practices. However, the present study did not quantify the dietary contribution, harvesting volume, or economic importance of these species; consequently, their role in food security and food sovereignty should be interpreted as a culturally relevant pattern rather than as a measured nutritional or socioeconomic effect.

### 3.3. Public Health and Safety Implications

The recorded remedies form part of household and community-based healthcare practices in the Guba District. Their continued use may reflect familiarity, cultural continuity, local availability, and the recognized knowledge of traditional healers. The 17 healers included in the survey reported broader medicinal plant knowledge than other participants, indicating their specialized role in maintaining and transmitting ethnomedicinal practices. However, the study did not collect systematic information on the frequency with which plant remedies were used instead of, before, or alongside biomedical treatment. The findings should therefore not be interpreted as demonstrating that traditional remedies replace formal healthcare. Rather, they document a locally important body of knowledge that may coexist with biomedical services and that requires careful evaluation of efficacy, interactions, dosage, and safety.

Several potentially toxic plants were included in the recorded pharmacopoeia. The leaves of *Atropa belladonna* and *Hyoscyamus niger* were reported for topical use against joint and rheumatic pain, while the leaves of *Bryonia aspera* and *Taxus baccata* and the underground organs of *Colchicum speciosum* were used topically for skin problems. *Chelidonium majus* was reported for both oral and topical applications, and *Peganum harmala* was used topically for wound healing and through the fumigation of burned aerial parts. Informants stated that they were aware that some of these plants required particular caution and associated safe use with preparation method, dose, plant part, collection season, and duration of application. Nevertheless, self-reported awareness does not establish toxicological safety. Several of these taxa contain potent bioactive or toxic constituents, and inappropriate preparation, dosage, ingestion, dermal exposure, or smoke inhalation may cause serious adverse effects. These records should therefore be understood as documentation of traditional practices rather than recommendations for unsupervised use. Toxicological assessment, clinically relevant dose evaluation, and clear community risk communication are needed, particularly for *A. belladonna*, *H. niger*, *T. baccata*, *C. speciosum*, *B. aspera*, *C. majus*, and *P. harmala*.

### 3.4. Study Limitations

Several limitations should be considered when interpreting the findings of this study. First, the information represents self-reported traditional knowledge and practices and should not be equated with clinically verified therapeutic effects. The purposive and snowball sampling strategy may have preferentially reached individuals recognized for their knowledge of medicinal plants; therefore, the sample cannot be considered statistically representative of the entire population of the Guba District. The predominance of male informants and pensioners, together with the unequal representation of occupational and ethnolinguistic groups and the small sizes of some occupational categories, may also have influenced the range and types of medicinal uses recorded. In particular, the predominance of male informants may have resulted in the under-documentation of knowledge traditionally maintained or more readily discussed by women, including certain gynecological, pediatric, household, and culinary–medicinal practices. The relatively high proportion of pensioners may also have emphasized the knowledge and experiences of older adults while providing less insight into plant-use practices among younger generations. These possibilities were not directly tested and should therefore be regarded as potential sampling effects rather than demonstrated differences. Consequently, the descriptive quantitative indices and group-level patterns characterize only the surveyed informants and should not be interpreted as population-level estimates. Furthermore, RFC and UV, as well as the numbers of medicinal taxa and use-reports, are derived from related components of the same dataset. Associations between these measures may therefore be mathematically expected and were not treated as independent inferential evidence in the revised analysis. Future studies based on stratified or probability-based sampling and more balanced group sizes are required to evaluate demographic, occupational, and geographical differences more robustly.

Although ethnolinguistic affiliation was recorded for each informant, the data were not originally structured as a complete community-specific presence/absence matrix linking every taxon and use-report to each ethnolinguistic group. Consequently, reliable Jaccard similarity indices and comparisons of community-specific plant repertoires could not be reconstructed without returning to the original interview records. The present revision therefore reports ethnolinguistic differences only in the breadth of knowledge and does not claim that the communities possess either shared or distinct medicinal floras. Future analysis based on fully disaggregated species-by-informant data is required to evaluate repertoire overlap and community-specific uses.

All interviews were conducted in Azerbaijani because the participants from the different ethnolinguistic communities were fluent in the language and used it for interethnic communication. Multilingual vernacular plant names were nevertheless recorded and are presented in the taxa table. However, conducting the interviews primarily in Azerbaijani may have limited the depth of culturally specific terminology and emic descriptions of illness and treatment. Future in-depth studies conducted directly in the respective minority languages, in cooperation with native speakers and local cultural experts, may reveal additional linguistic and cultural dimensions of ethnomedicinal knowledge.

The interviews were designed as focused semi-structured encounters lasting 10–30 min because many participants were engaged in farming, shepherding, and other time-demanding occupations and were not willing or able to participate in longer sessions. Although this approach enabled broad coverage of 260 informants across 40 settlements, the relatively short interview duration may have limited the collection of detailed ethnographic narratives, preparation procedures, dosage information, and culturally specific explanations of therapeutic practices. The present study should therefore be regarded as a broad district-level ethnobotanical survey rather than an in-depth ethnographic investigation.

The study also did not directly quantify harvesting intensity, market demand, or population-level pressures on the recorded medicinal species. Moreover, the cross-sectional design and the limited representation of younger informants do not allow direct conclusions regarding the rate of intergenerational knowledge transmission. Future research should combine multilingual and community-centered ethnographic documentation with ecological assessments of harvesting pressure, sustainable-use initiatives, phytochemical and pharmacological investigation, and safety evaluation of culturally important and potentially toxic medicinal plants.

### 3.5. Community-Centered Implementation and Knowledge Return

A community-centered implementation strategy should begin by returning the documented knowledge to the participating settlements in accessible and locally meaningful forms. Subject to community review and consent, the study database could be used to prepare a concise multilingual guide presenting verified botanical names, voucher references, field photographs, where available, recorded vernacular names, plant parts used, and locally reported applications in Azerbaijani and, where available, Lezgian, Tat, Khinalug, Kryts, Jek/Buduq, and Juhuri. Printed copies and simplified educational materials could be shared through village schools, municipal offices, cultural centers, and local museums. School- and village-based documentation activities involving elders, healers, teachers, and younger residents could facilitate the sharing and documentation of ethnobotanical knowledge across generations while contributing to the preservation of endangered minority-language plant terminology. Community validation would be essential before dissemination to prevent linguistic errors, inappropriate attribution, or the disclosure of knowledge that participants consider sensitive.

Practical implementation should also involve cooperation with local institutions. Collaboration with Shahdag National Park and village authorities could support species-specific guidance on sustainable collection, with priority given to nationally listed taxa and species harvested for roots, bulbs, tubers, or bark. In Khinalig, Laza, and other tourism-oriented settlements, locally approved ethnobotanical information could be incorporated into guided walks, visitor materials, or community-based tourism initiatives, provided that these activities do not encourage collection of threatened or toxic plants. Cooperation with primary healthcare professionals could focus on risk communication concerning potentially toxic taxa, contraindications, herb–drug interactions, and the importance of seeking medical care for serious conditions. Any public-facing material should clearly distinguish informant-reported use from clinically validated treatment and should be developed with community participation, appropriate acknowledgment, and respect for collective knowledge ownership.

## 4. Materials and Methods

### 4.1. Study Area

**Figure 4:**
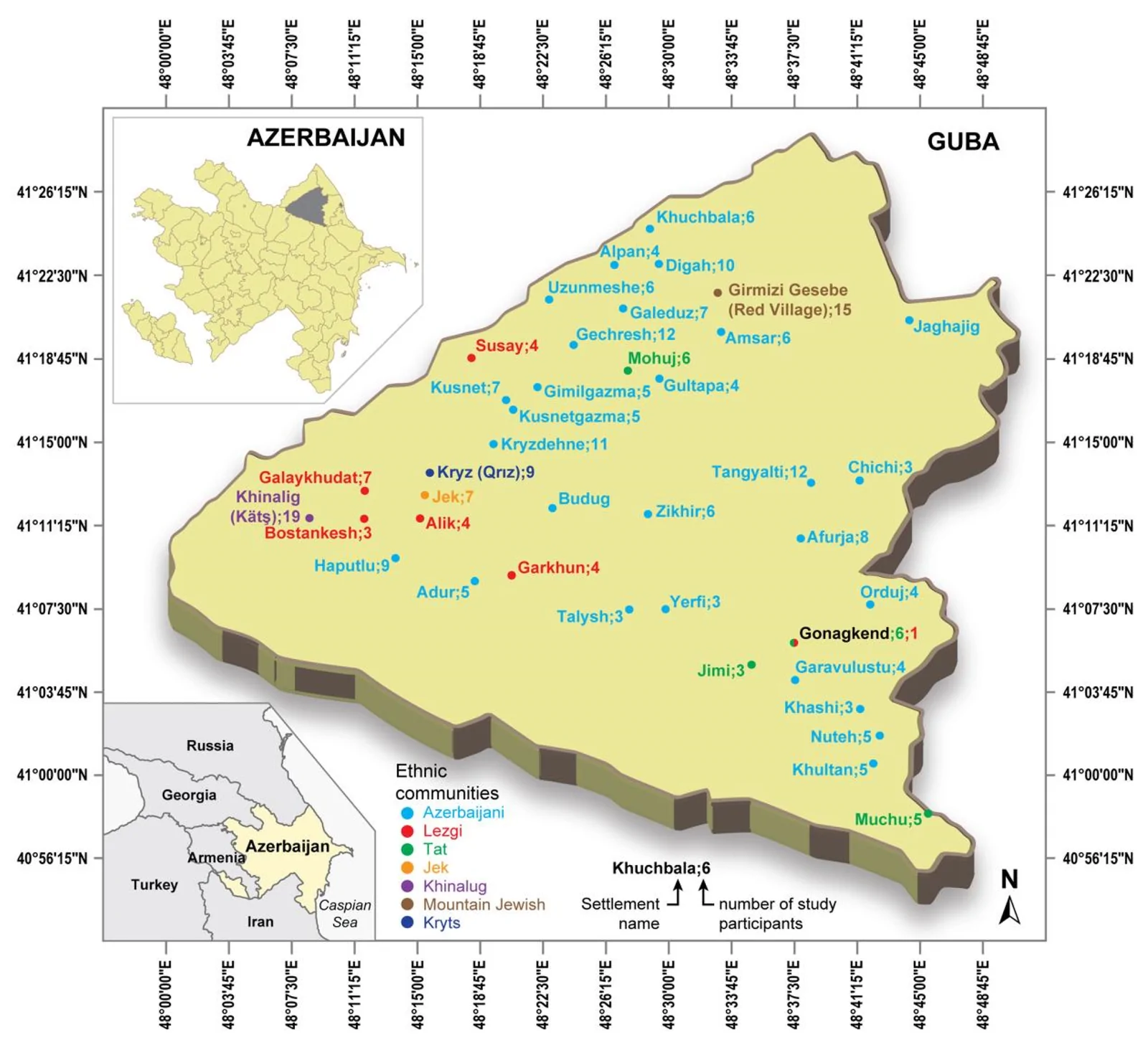
Locations of the 40 villages surveyed in the Guba District.

The present ethnopharmacological study was conducted in the Guba District (Quba Rayonu), located on the northeastern slopes of the Greater Caucasus in northeastern Azerbaijan (geographic centre: 41°21′55″ N, 48°31′35″ E; Figure 4). The district borders the Akhty District of the Republic of Dagestan (Russian Federation) to the north and the Azerbaijani districts of Gusar, Gabala, Ismayilli, Shabran, and Siyazan. Covering an area of approximately 2610 km^2^, the district encompasses four major ecological zones—lowland, foothill, mid-mountain, and high-mountain—with elevations ranging from approximately 200 to 4243 m above sea level. This pronounced altitudinal gradient creates considerable ecological and vegetation diversity and supports a rich flora containing numerous medicinal plant species. A substantial part of the district lies within Shahdag National Park, contributing to the conservation of the region’s biological diversity.

The relief of the district comprises foothill plains together with mid- and high-mountain landscapes. The Yarimsa, Mikhtokan, Yerfi, and Tanga mountain ranges branch from the Lateral Range, which extends parallel to the Main Caucasus Range. Climatic conditions vary considerably with altitude. The lowland and foothill zones are characterized by a moderately warm climate with relatively even precipitation throughout the year, whereas the high-mountain areas experience a cold mountain-tundra climate. Mean January temperatures range from −14 to −2 °C, while mean July temperatures vary between 5 and 23 °C. Annual precipitation increases with elevation, ranging from approximately 500 mm in the lowlands to 900 mm in the mountainous areas.

Alpine and subalpine meadows occupy extensive areas of the mountainous landscape, whereas broad-leaved forests characterize the lower mountain belts. Beech, oak, and hornbeam are among the dominant forest-forming trees in the mountain forests of Azerbaijan [44].

The local economy is based primarily on agriculture and animal husbandry. The district has a culturally diverse population comprising Azerbaijanis, Lezgins, Tats, Khinalugs, Kryts, Jeks, and Mountain Jews. Azerbaijanis constitute the largest ethnic group in the Guba District, accounting for 75.29% of the district population according to the 2019 population census [45]. Azerbaijani also serves as the principal language of wider communication in the district [46]. Lezgins are concentrated in northeastern Azerbaijan, including the Guba region, and speak Lezgi, a Northeast Caucasian language. Lezgi remains widely used within the community alongside Azerbaijani, and the population is traditionally Sunni Muslim [46]. Tats of northeastern Azerbaijan speak Tat, an Iranian language. Sociolinguistic studies indicate considerable variation among settlements and a progressive decline in Tat fluency across generations [47]. Khinalugs are a high-mountain community centered on Khinalug village and speak the indigenous Khinalug language. The community is predominantly Sunni Muslim, sheep breeding has traditionally been a major livelihood, and increasing contact with Azerbaijani is creating pressure on intergenerational language transmission [48]. Kryts originate from the highlands east of Mount Shahdagh in the Guba region and speak Kryts, a Lezgic language with several dialects. Livestock breeding has traditionally been important, while relocation from mountain villages to the plains has contributed to substantial language shift toward Azerbaijani [49]. Jeks (Cek) are a small high-mountain community associated with Cek village and speak a variety belonging to the Kryts dialect continuum. Pastoralism has traditionally been central to local livelihoods, while increasing use of Azerbaijani accompanies broader sociolinguistic change [49]. Mountain Jews are particularly associated with Qırmızı Qəsəbə (Krasnaya Sloboda) near Guba and traditionally speak Juhuri (Judeo-Tat), an Iranian Jewish language. The community is characterized by extensive multilingualism, although Juhuri continues to have an important role in local community life [50]. Religious affiliation was not recorded at the individual level in the present survey; therefore, the community-level information above is based on published ethnographic and sociolinguistic sources and should not be interpreted as describing the religious identity of individual informants. To capture the ecological and cultural breadth of the district, field investigations were conducted in 40 settlements distributed across the principal ecological zones of the study area (Table 10).

### 4.2. Ethical Considerations

The study protocol was reviewed and approved by the Local Ethics Committee for Fundamental Scientific Research of Azerbaijan Medical University (protocol code AMU/FSR-LEC-2024-01/01, approved on 6 May 2024) before the commencement of fieldwork. Prior to each interview, the objectives and procedures of the study were explained to all participants, and oral informed consent was obtained. Participation was entirely voluntary, and respondents were informed of their right to decline participation or withdraw from the interview at any stage without consequences. All personal information was anonymized before data analysis and publication. The study was conducted in accordance with the principles of the International Society of Ethnobiology Code of Ethics [51] and the recommendations for ethnopharmacological field studies proposed by Weckerle et al. [8] and Heinrich et al. [52]. No personally identifiable information is presented in this study.

### 4.3. Sampling and Informants

Informants were recruited through purposive selection of initial respondents followed by snowball sampling [53,54,55]. Initial respondents in each village were identified through locally appropriate contacts, including community leaders, knowledgeable residents, and other trusted individuals familiar with the local community. These respondents subsequently recommended additional individuals recognized for their knowledge of traditional medicinal plants, thereby establishing multiple independent recruitment chains across the study area. A total of 260 informants from 40 villages were interviewed. The final sample size was determined by village accessibility, informant availability, willingness to participate, and the objective of documenting traditional medicinal plant knowledge across all ecological zones and major ethnic communities of the district. The inclusion criteria were: (i) age ≥ 18 years; (ii) permanent residence in the Guba District; and (iii) voluntary participation following oral informed consent.

**Table 10:** Settlements of the Guba District surveyed in this study.

**A. Geographical and ecological characteristics**								
**No.**	**Settlement** **name** **(official** **Azerbaijani)**	**Standardized** **Latin name/** **local name**	**GPS coordinates**	**Altitude** **(m a.s.l.)**	**Ecological zone**	**Dominant vegetation**		**Population**
1	Adur	Adur	41°07′32″ N, 48°17′29″ E	1200	Middle mountain-forest belt	Oriental beech, oak, and hornbeam forests		537
2	Afurca	Afurja	41°09′39″ N, 48°36′56″ E	970	Lower mountain-forest belt	Broadleaved mountain forest dominated by oak and hornbeam		693
3	Alpan	Alpan	41°23′17″ N, 48°25′49″ E	760	Lower mountain-forest belt	Broadleaved mountain forest dominated by oak and hornbeam		3837
4	Amsar	Amsar	41°19′58″ N, 48°32′11″ E	1100	Lower mountain-forest belt	Broadleaved mountain forest dominated by oak and hornbeam		2629
5	Bostankeş	Bostankesh	41°10′38″ N, 48°10′53″ E	1800	High-mountain subalpine meadows	Subalpine tall-herb vegetation and mountain meadows		100
6	Buduq	Budug	41°11′10″ N, 48°22′07″ E	1760	Upper mountain-forest and forest–meadow transition	Upper mountain forest, open woodland, and meadow mosaic		300
7	Cağacıq	Jaghajig	41°20′33″ N, 48°43′25″ E	300	Foothill and low-mountain zone	Forest-steppe, shrubland, and cultivated land		492
8	Cek	Jek	41°11′49″ N, 48°14′30″ E	1643	Upper mountain-forest and forest–meadow transition	Upper mountain forest, open woodland, and meadow mosaic		328
9	Cimi	Jimi	41°03′21″ N, 48°34′00″ E	1282	Middle mountain-forest belt	Oriental beech, oak, and hornbeam forests		1209
10	Çiçi	Chichi	41°12′33″ N, 48°40′27″ E	541	Foothill and low-mountain zone	Forest-steppe, shrubland, and cultivated land		814
11	Digah	Digah	41°23′20″ N, 48°28′28″ E	674	Lower mountain-forest belt	Broadleaved mountain forest dominated by oak and hornbeam		1725
12	Əlik	Alik	41°10′39″ N, 48°14′13″ E	1679	Upper mountain-forest and forest–meadow transition	Upper mountain forest, open woodland, and meadow mosaic		372
13	Gültəpə	Gultapa	41°17′38″ N, 48°28′30″ E	900	Lower mountain-forest belt	Broadleaved mountain forest dominated by oak and hornbeam		545
14	Haputlu	Haputlu	41°08′40″ N, 48°12′45″ E	1600	Upper mountain-forest and forest–meadow transition	Upper mountain forest, open woodland, and meadow mosaic		505
15	Xaltan	Khultan	40°58′24″ N, 48°41′16″ E	1088	Lower mountain-forest belt	Broadleaved mountain forest dominated by oak and hornbeam		624
16	Xaşı	Khashi	41°01′08″ N, 48°40′29″ E	1300	Middle mountain-forest belt	Oriental beech, oak, and hornbeam forests		160
17	Xınalıq	Khinalig (Khinalug: Kätş)	41°10′40″ N, 48°07′37″ E	2350	High-mountain alpine meadows and rocky habitats	Alpine meadow, low-growing grassland, and rocky vegetation		2075
18	Xuçbala	Khuchbala	41°25′05″ N, 48°27′56″ E	1600	Upper mountain-forest and forest–meadow transition	Upper mountain forest, open woodland, and meadow mosaic		1480
19	Küsnət	Kusnet	41°16′34″ N, 48°19′21″ E	1100	Lower mountain-forest belt	Broadleaved mountain forest dominated by oak and hornbeam		285
20	Küsnətqazma	Kusnetgazma	41°16′ N, 48°17′ E	1300	Middle mountain-forest belt	Oriental beech, oak, and hornbeam forests		466
21	Qalayxudat	Galaykhudat	41°12′02″ N, 48°10′55″ E	1900	High-mountain subalpine meadows	Subalpine tall-herb vegetation and mountain meadows		404
22	Qaravülüstü	Garavulustu	41°02′35″ N, 48°36′36″ E	1050	Lower mountain-forest belt	Broadleaved mountain forest dominated by oak and hornbeam		176
23	Qarxun	Garkhun	41°07′49″ N, 48°19′41″ E	1685	Upper mountain-forest and forest–meadow transition	Upper mountain forest, open woodland, and meadow mosaic		586
24	Qəçrəş	Gechresh	41°19′19″ N, 48°23′23″ E	859	Lower mountain-forest belt	Broadleaved mountain forest dominated by oak and hornbeam		2316
25	Qələdüz	Galeduz	41°21′08″ N, 48°26′20″ E	750	Lower mountain-forest belt	Broadleaved mountain forest dominated by oak and hornbeam		779
26	Qımılqazma	Gimilgazma	41°15′ N, 48°18′ E	900	Lower mountain-forest belt	Broadleaved mountain forest dominated by oak and hornbeam		500
27	Qırmızı qəsəbə	Girmizi Gesebe (Red Village)	41°21′55″ N, 48°31′59″ E	800	Lower mountain-forest belt	Broadleaved mountain forest dominated by oak and hornbeam		3252
28	Qrız	Kryz (Kryts: Qrız)	41°12′56″ N, 48°14′48″ E	2016	High-mountain subalpine meadows	Subalpine tall-herb vegetation and mountain meadows		274
29	Qonaqkənd	Gonagkend	41°04′27″ N, 48°36′32″ E	1076	Lower mountain-forest belt	Broadleaved mountain forest dominated by oak and hornbeam		1615
30	Qrızdəhnə	Kryzdehne	41°14′22″ N, 48°18′36″ E	1400	Middle mountain-forest belt	Oriental beech, oak, and hornbeam forests		164
31	Möhüc	Mohuj	41°18′02″ N, 48°26′37″ E	1037	Lower mountain-forest belt	Broadleaved mountain forest dominated by oak and hornbeam		1329
32	Muçu	Muchu	40°55′53″ N, 48°44′32″ E	1149	Lower mountain-forest belt	Broadleaved mountain forest dominated by oak and hornbeam		492
33	Nütəh	Nuteh	40°59′48″ N, 48°41′39″ E	1015	Lower mountain-forest belt	Broadleaved mountain forest dominated by oak and hornbeam		145
34	Ördüc	Orduj	41°06′22″ N, 48°41′05″ E	1118	Lower mountain-forest belt	Broadleaved mountain forest dominated by oak and hornbeam		208
35	Susay	Susay	41°18′40″ N, 48°17′17″ E	1237	Middle mountain-forest belt	Oriental beech, oak, and hornbeam forests		123
36	Talış	Talysh	41°06′07″ N, 48°26′42″ E	1432	Middle mountain-forest belt	Oriental beech, oak, and hornbeam forests		161
37	Təngəaltı	Tangyalti	41°12′26″ N, 48°37′33″ E	702	Lower mountain-forest belt	Broadleaved mountain forest dominated by oak and hornbeam		705
38	Uzunmeşə	Uzunmeshe	41°21′35″ N, 48°21′55″ E	928	Lower mountain-forest belt	Broadleaved mountain forest dominated by oak and hornbeam		232
39	Yerfi	Yerfi	41°06′08″ N, 48°28′52″ E	1300	Middle mountain-forest belt	Oriental beech, oak, and hornbeam forests		470
40	Zıxır	Zikhir	41°10′52″ N, 48°27′49″ E	790	Lower mountain-forest belt	Broadleaved mountain forest dominated by oak and hornbeam		116
**B. Participant and ethnolinguistic profile**								
**No.**	**Settlement**	**Self-identified community of participants**	**Language and Glottolog code**	**Participants, *n***	**Female/** **Male**	**Age range**	**Healers, *n***	**Main occupational profile**
1	Adur	Azerbaijani (*n* = 5)	Azerbaijani [azer1255]	5	2/3	31–65	1	Teacher (2); Homemaker (1); Municipal representative (1)
2	Afurja	Azerbaijani (*n* = 8)	Azerbaijani [azer1255]	8	2/6	39–89	0	Pensioners (2); Farmer (2); Homemaker (1)
3	Alpan	Azerbaijani (*n* = 4)	Azerbaijani [azer1255]	4	0/4	29–72	0	Teacher (1); Pensioners (1); Forest ranger (1)
4	Amsar	Azerbaijani (*n* = 6)	Azerbaijani [azer1255]	6	3/3	28–87	2	Homemaker (2); Teacher (1); Municipal representative (1)
5	Bostankesh	Lezgi (*n* = 3)	Lezgian [lezg1247]	3	0/3	39–42	0	Shepherds (2); Farmer (1)
6	Budug	Azerbaijani (*n* = 10)	Azerbaijani [azer1255]	10	3/7	38–84	0	Pensioners (3); Shepherds (2); Homemaker (1)
7	Jaghajig	Azerbaijani (*n* = 4)	Azerbaijani [azer1255]	4	3/1	32–75	0	Farmer (2); Homemaker (1); Teacher (1)
8	Jek	Jek (*n* = 7)	Jek [dzhe1238]	7	3/4	28–92	1	Homemaker (2); Pensioners (2); Teacher (1)
9	Jimi	Tat (*n* = 3)	Tat [tati1241]	3	1/2	50–73	0	Homemaker (1); Teacher (1); Shepherds (1)
10	Chichi	Azerbaijani (*n* = 3)	Azerbaijani [azer1255]	3	1/2	49–61	0	Teacher (1); Municipal representative (1); Forest ranger (1)
11	Digah	Azerbaijani (*n* = 10)	Azerbaijani [azer1255]	10	7/3	45–80	0	Homemaker (3); Pensioners (3); Teacher (2)
12	Alik	Lezgi (*n* = 4)	Lezgian [lezg1247]	4	2/2	50–75	0	Homemaker (1); Local executive representative (1); Shepherds (1)
13	Gultapa	Azerbaijani (*n* = 4)	Azerbaijani [azer1255]	4	2/2	40–78	1	Homemaker (2); Farmer (2)
14	Haputlu	Azerbaijani (*n* = 9)	Azerbaijani [azer1255]	9	3/6	41–79	1	Pensioners (3); Homemaker (2); Teacher (1)
15	Khultan	Azerbaijani (*n* = 5)	Azerbaijani [azer1255]	5	2/3	70–76	0	Farmer (3); Homemaker (1); Pensioners (1)
16	Khashi	Azerbaijani (*n* = 3)	Azerbaijani [azer1255]	3	1/2	75–90	0	Pensioners (2); Farmer (1)
17	Khinalig (Khinalug: Kätş)	Khinalug (*n* = 19)	Khinalug [khin1240]	19	6/13	43–87	2	Pensioners (6); Shepherds (4); Homemaker (3)
18	Khuchbala	Azerbaijani (*n* = 6)	Azerbaijani [azer1255]	6	2/4	45–78	1	Homemaker (2); Farmer (2); Municipal representative (1)
19	Kusnet	Azerbaijani (*n* = 7)	Azerbaijani [azer1255]	7	3/4	56–90	0	Pensioners (3); Homemaker (1); Teacher (1)
20	Kusnetgazma	Azerbaijani (*n* = 5)	Azerbaijani [azer1255]	5	2/3	33–73	0	Homemaker (1); Teacher (1); Municipal representative (1)
21	Galaykhudat	Lezgi (*n* = 7)	Lezgian [lezg1247]	7	5/2	58–76	1	Homemaker (2); Teacher (2); Municipal representative (1)
22	Garavulustu	Azerbaijani (*n* = 4)	Azerbaijani [azer1255]	4	2/2	41–83	0	Homemaker (1); Teacher (1); Local executive representative (1)
23	Garkhun	Lezgi (*n* = 4)	Lezgian [lezg1247]	4	0/4	40–70	0	Pensioners (1); Forest ranger (1); Shepherds (1)
24	Gechresh	Azerbaijani (*n* = 12)	Azerbaijani [azer1255]	12	5/7	35–80	1	Teacher (3); Pensioners (3); Farmer (3)
25	Galeduz	Azerbaijani (*n* = 7)	Azerbaijani [azer1255]	7	1/6	29–83	0	Teacher (2); Pensioners (2); Farmer (2)
26	Gimilgazma	Azerbaijani (*n* = 5)	Azerbaijani [azer1255]	5	1/4	29–80	1	Teacher (3); Pensioners (2)
27	Girmizi Gesebe (Red Village)	Mountain Jewish (*n* = 15)	Juhuri/Judeo-Tat [jude1256]	15	5/10	29–87	0	Pensioners (10); Teacher (2); Homemaker (1)
28	Kryz (Kryts: Qrız)	Kryts (*n* = 9)	Kryts [kryt1240]	9	3/6	35–85	0	Homemaker (2); Pensioners (2); Shepherds (2)
29	Gonagkend	Tat (*n* = 6); Lezgi (*n* = 1)	Tat [tati1241]; Lezgian [lezg1247]	7	2/5	33–77	0	Farmer (3); Homemaker (1); Local executive representative (1)
30	Kryzdehne	Azerbaijani (*n* = 11)	Azerbaijani [azer1255]	11	4/7	30–92	0	Teacher (5); Pensioners (5); Farmer (1)
31	Mohuj	Tat (*n* = 6)	Tat [tati1241]	6	2/4	57–91	1	Pensioners (2); Homemaker (1); Teacher (1)
32	Muchu	Tat (*n* = 5)	Tat [tati1241]	5	3/2	57–63	0	Homemaker (1); Teacher (1); Municipal representative (1)
33	Nuteh	Azerbaijani (*n* = 5)	Azerbaijani [azer1255]	5	0/5	39–87	0	Pensioners (3); Teacher (2)
34	Orduj	Azerbaijani (*n* = 4)	Azerbaijani [azer1255]	4	1/3	49–88	1	Teacher (2); Pensioners (2)
35	Susay	Lezgi (*n* = 4)	Lezgian [lezg1247]	4	3/1	29–89	0	Homemaker (1); Teacher (1); Municipal representative (1)
36	Talysh	Azerbaijani (*n* = 3)	Azerbaijani [azer1255]	3	0/3	29–74	0	Pensioners (2); Teacher (1)
37	Tangyalti	Azerbaijani (*n* = 12)	Azerbaijani [azer1255]	12	0/12	73–89	1	Pensioners (12)
38	Uzunmeshe	Azerbaijani (*n* = 6)	Azerbaijani [azer1255]	6	0/6	45–89	1	Pensioners (4); Teacher (2)
39	Yerfi	Azerbaijani (*n* = 3)	Azerbaijani [azer1255]	3	0/3	73–88	0	Pensioners (3)
40	Zikhir	Azerbaijani (*n* = 6)	Azerbaijani [azer1255]	6	0/6	68–86	1	Pensioners (6)

**Notes:** Coordinates were transcribed from the authors’ field table. Seconds were not available for Kusnetgazma and Gimilgazma and were therefore not inferred. Ethnolinguistic affiliations are based on participants’ self-identification in the authors’ field records; counts in mixed settlements are shown separately. Language names and Glottolog codes correspond to the reported ethnolinguistic affiliations. All interviews were conducted in Azerbaijani, the shared language of interethnic communication in the study area. Religious affiliation was not included in the questionnaire and is therefore not reported.

Key informants were identified based on their recognized expertise and long-standing experience in the traditional use of medicinal plants. Because the sample sizes of the ethnolinguistic communities were highly unequal, formal comparative analyses among these communities were not performed. Species-level quantitative ethnobotanical indices were calculated using the pooled dataset, whereas healer status, gender, educational level, settlement, and ecological zone were examined separately using descriptive summaries of the numbers of taxa and use-reports reported per informant.

### 4.4. Field Research and Interview Protocol

Fieldwork was conducted during six expeditions undertaken in the spring, summer, and autumn of 2024 and 2025, with three expeditions completed each year. The surveys were scheduled to coincide with different flowering and vegetation stages of medicinal plant species to facilitate accurate field identification and specimen collection. Repeated visits to villages during different seasons also enabled the documentation of seasonal variation in medicinal plant availability and the recording of traditional knowledge relating to species collected at different times of the year.

#### 4.4.1. Interview Format and Content

Individual semi-structured interviews were conducted with each respondent using a standardized ethnobotanical questionnaire developed for this study. The questionnaire comprised the following sections: (i) demographic characteristics, including age, sex, ethnic affiliation, education level, occupation, and duration of residence in the village; (ii) local names of medicinal plants and, where available, corresponding voucher specimens; (iii) plant parts used; (iv) preparation methods; (v) routes of administration; (vi) ailments, symptoms, or health conditions treated; (vii) sources of traditional knowledge; and (viii) frequency and context of use. No precompiled plant checklist was shown to the participants. Medicinal plants were elicited through open-ended questions in which informants were asked to name the plants they knew or used, followed by probing questions concerning local names, plant parts, preparation, administration, and reported uses. Plant identification was subsequently supported through field observation and the collection of voucher specimens with knowledgeable informants. Interviews typically lasted 10–30 min. The relatively short interview duration reflected the practical conditions of fieldwork and the preferences of the participants. In many villages, residents were not willing or able to participate in extended interviews. As participation was entirely voluntary, the research team respected each informant’s preferred level and duration of engagement. Moreover, many participants were engaged in farming, shepherding, and other physically demanding occupations and could not remain away from their daily work for prolonged periods. The interviews were therefore designed as focused semi-structured encounters lasting up to 30 min. Information was recorded either through audio recording or detailed written notes, according to the respondent’s preference and consent.

All interviews were conducted in Azerbaijani, which functions as the principal language of interethnic communication in the Guba District. Participants belonging to the Lezgin, Tat, Khinalug, Kryts, Jek, and Mountain Jewish communities were fluent Azerbaijani speakers and were comfortable conducting the interviews in Azerbaijani; therefore, interpreters were not required. The field team conducted the interviews in Azerbaijani and did not claim professional proficiency in all of the minority languages represented in the study; minority-language plant names were recorded as provided by the informants. Where available, informants also provided plant names used in their respective community languages. These vernacular names were recorded together with the corresponding language and are presented in the principal taxa table.

#### 4.4.2. Plant Collection and Taxonomic Identification

Field investigations were conducted together with local respondents to document traditional medicinal uses and to collect representative specimens of the plants cited during the interviews. The collected specimens were identified under the supervision of Javanshir Isayev (Department of Pharmacognosy, Azerbaijan Medical University) through detailed morphological examination of the collected material and comparison with authenticated taxonomic and photographic resources.

Voucher specimens were prepared, assigned unique voucher codes, and deposited in the departmental herbarium collection of the Department of Pharmacognosy, Azerbaijan Medical University, Baku, Azerbaijan. This institutional teaching and research collection is not currently registered in Index Herbariorum and therefore does not have an official Index Herbariorum acronym. The voucher specimens are available for consultation by researchers upon reasonable request and prior arrangement with the Department of Pharmacognosy. Voucher codes follow the format AZE-IJ-001 to AZE-IJ-204, in which “AZE” denotes Azerbaijan, “IJ” denotes the initials of the collector, Javanshir Isayev, and the final three-digit number represents the sequential specimen number. The voucher codes corresponding to the 193 taxa included in the principal dataset are provided in the main taxa table, while those corresponding to the 11 additional localized taxa are presented in the separate additional-taxa table.

All plant specimens were collected and deposited within Azerbaijan, and no transboundary transfer of genetic resources occurred; therefore, no separate access and benefit-sharing statement under the Nagoya Protocol was required for this study. Accepted scientific names, author citations, and family classifications were verified against the World Flora Online Plant List, version 2026-06. Synonyms were replaced with the corresponding accepted names, while taxa classified by WFO as unchecked or not represented in the consulted backbone were retained with an explanatory note rather than being assigned an unsupported taxonomic status [56].

### 4.5. Data Management and Classification of Use-Reports

All data collected during the field surveys were entered, organized, and managed using Microsoft Excel 2019 (Microsoft Corporation, Redmond, WA, USA). Following Weckerle et al. [8], a use-report (UR)—defined as a single record in which one informant reported one use of one plant species for a specific disease or health condition—was treated as the basic unit of analysis.

The principal dataset comprised 7385 informant–taxon citations and 8682 use-reports for 193 medicinal plant taxa. The family-level use-report totals presented in Table 3 and the taxon-level ΣU values presented in Table 4 both sum to the same grand total of 8682 use-reports. Information reproduced in the Supplementary Materials represents a more detailed presentation of records already included in the principal dataset and was not counted as additional use-reports. The 11 rare, locality-specific taxa presented separately in Table 5 were excluded from this grand total and from the principal quantitative analyses.

Quantitative ethnobotanical indices, including Frequency of Citation (FC), Relative Frequency of Citation (RFC), Use Value (UV), Fidelity Level (FL), Informant Consensus Factor (ICF), and Family Use Value (FUV), were calculated using Microsoft Excel 2019. Informants’ original descriptions of ailments, symptoms, and intended therapeutic purposes were recorded first and retained as emic use descriptions in the principal taxa table. For quantitative synthesis, semantically related reports were subsequently grouped into ten broad, mutually exclusive health-condition categories. ICD-11 was consulted only as a secondary analytical reference to support consistent organization and terminology; it was not used to replace local illness concepts or to reinterpret participants’ descriptions as clinically verified diagnoses [57]. Each use-report was assigned to only one broad category to prevent duplicate counting across diseases, symptoms, and pharmacological actions.

Eleven taxa cited by only one to four informants and restricted to a single settlement were classified as rare, locality-specific records. The principal quantitative dataset therefore comprised taxa with FC ≥ 8; no recorded taxon had an FC between 5 and 7. These taxa were excluded from the principal index-based comparisons because their very low citation frequencies did not permit meaningful quantitative comparison. However, they were retained in the ethnobotanical inventory and reported separately to avoid the loss of potentially culturally and geographically specific medicinal plant knowledge.

To examine variation in ethnobotanical knowledge within the surveyed sample, informants were grouped according to healer status, gender, educational level, settlement, ecological zone, and self-identified ethnolinguistic affiliation. For each group, the number of informants and the median and interquartile range of the numbers of medicinal plant taxa and use-reports provided per informant were calculated. Because purposive and snowball sampling was used and several groups were small or unevenly represented, these comparisons were treated as descriptive patterns within the surveyed sample rather than as population-level estimates. No inferential tests were applied to the ethnolinguistic groups.

### 4.6. Quantitative Ethnobotanical Indices

The cultural prominence of medicinal plant species and the level of agreement among informants were assessed using six standard quantitative ethnobotanical indices.

Use Value (UV)

Use Value was calculated for each species according to Phillips and Gentry [58].

UV = ΣU_i_/N

where U_i_ is the number of use-reports mentioned by informant i for a given species and N is the total number of informants interviewed. Higher UV values indicate greater cultural prominence of a species within the surveyed population.

Frequency of Citation (FC)

Frequency of Citation was calculated as the absolute number of informants reporting at least one use of a given species [59].

Relative Frequency of Citation (RFC)

Relative Frequency of Citation was calculated according to Tardío and Pardo-de-Santayana [60].

RFC = FC/N 0 < RFC ≤ 1

where FC is the number of informants citing a species and N is the total number of informants interviewed. RFC normalizes FC by sample size and facilitates comparison among species and, where sampling designs are comparable, across studies.

Fidelity Level (FL)

Fidelity Level expresses the percentage of informants citing a given species for a particular reported use or condition and was calculated according to Friedman et al. [61].

FL (%) = (N_p_/N_s_) × 100

where N_p_ is the number of informants citing a species for a specific reported use or condition and N_s_ is the total number of informants citing that species for any medicinal use. FL was calculated for each reported species–use combination. High FL values indicate that informant citations for a species are strongly concentrated on a particular therapeutic purpose.

Informant Consensus Factor (ICF)

The Informant Consensus Factor measures agreement among informants regarding the taxa used for a given disease or health-condition subcategory and was calculated according to Trotter and Logan [62]:

ICF = (N_ur_ − N_t_)/(N_ur_ − 1)

where N_ur_ is the total number of use-reports for a given subcategory and N_t_ is the number of medicinal plant taxa used within that subcategory. ICF values range from 0 (no agreement) to 1 (complete agreement). High ICF values indicate that relatively few taxa account for a large number of use-reports, suggesting a shared therapeutic tradition.

Family Use Value (FUV)

Family Use Value was calculated according to Hoffman and Gallaher [36] to assess the average ethnobotanical importance of the species recorded within each botanical family:

FUV = ΣUV_s_/n_s_

where ΣUV_s_ is the sum of the UV values of all species belonging to a given family and n_s_ is the number of species recorded within that family. Higher FUV values indicate greater average ethnobotanical importance of the recorded species within the family.

### 4.7. Descriptive Data Analysis

The number of medicinal plant species and use-reports recorded for each informant was summarized using descriptive statistics. Counts and percentages were used to describe the demographic characteristics of the informants, while medians and interquartile ranges were used to summarize the number of medicinal plant species reported across occupational and other demographic groups. Because the informants were recruited through purposive and snowball sampling and because several demographic and occupational groups were small and unequal in size, between-group patterns were evaluated descriptively rather than through population-level inferential testing. The quantitative ethnobotanical indices were calculated as described above and were used to characterize the cultural prominence, diversity of use, and level of agreement associated with the documented medicinal plants.

### 4.8. Conservation Status Assessment

The national conservation status of all documented medicinal plant species was assessed using the third edition of the Red Book of the Republic of Azerbaijan (2023) and its accompanying Pink List of plant species [32]. Each recorded taxon was checked against these national references, and the resulting information was used to identify medicinal plants of conservation significance within the documented flora.

## 5. Conclusions

This study provides comprehensive documentation of medicinal plant knowledge in the Guba District of Azerbaijan, recording 193 species from 59 botanical families used in local traditional medicine. The predominance of herbaceous species and the frequent use of aerial plant parts reflect the reliance of local practices on readily available plant resources. Quantitative ethnobotanical analyses identified *Rosa tomentosa*, *Mentha aquatica*, *Origanum vulgare*, *Hippophae rhamnoides*, *Glycyrrhiza glabra*, *Inula helenium*, and *Anethum graveolens* as particularly prominent within the surveyed communities, while high ICF values indicated substantial agreement among informants across therapeutic categories. The documented medicinal uses were concentrated mainly on respiratory, digestive, genitourinary, dermatological, and cardiovascular conditions. Several of the reported species also have existing phytochemical or pharmacological evidence supporting their further investigation. Descriptive differences among occupational groups suggest that livelihood-related contact with local vegetation may contribute to variation in ethnobotanical knowledge; however, this observation requires confirmation through studies based on larger, more balanced, and probability-based samples.

Beyond their medicinal applications, the documented plants contributed to food, beekeeping, animal husbandry, and other household practices, emphasizing the broader biocultural importance of plant resources in Guba. At the same time, the inclusion of 24 medicinal species in the Red Book or Pink List of the Republic of Azerbaijan highlights the need to balance traditional use with sustainable harvesting and biodiversity conservation. Overall, the findings provide a baseline for preserving local ethnomedicinal knowledge and prioritizing culturally important species for targeted phytochemical, pharmacological, and safety studies. Beyond scientific investigation, the documented knowledge should be returned to the participating communities through community-validated multilingual educational materials, documentation initiatives involving both older and younger community members, cooperation with Shahdag National Park on sustainable harvesting, and locally appropriate public-health and community-tourism activities. Further experimental and clinical research is required before the reported traditional uses can be translated into evidence-based therapeutic applications.

## Figures and Tables

**Table 1:** Demographic characteristics of residents who participated in the survey (*n* = 260).

Parameter	Category	Count	%
Gender	Female	85	32.7
	Male	175	67.3
Age Group	28–40	31	11.9
	41–50	36	13.8
	51–60	47	18.1
	61–70	34	13.1
	71–80	77	29.6
	81–92	35	13.5
Education Level	Secondary education	183	70.4
	Incomplete secondary education	5	1.9
	Higher education	72	27.7
Occupation	Homemaker	36	13.8
	Teacher	46	17.7
	Executive representative	8	3.1
	Municipal representative	18	6.9
	Pensioner	91	35.0
	Forest ranger	9	3.5
	Shepherd	17	6.5
	Farmer	35	13.5

Note: Among the 260 respondents, 17 were identified as key informants based on their recognized expertise and long-standing experience in the traditional use of medicinal plants. Most were well-known traditional healers within their respective communities.

**Table 2:** Descriptive comparison of the numbers of medicinal plant taxa and use-reports reported per informant according to healer status, gender, educational level, and ecological zone.

Grouping Variable	Category	*n*	Reported Species, Median (IQR)	Use-Reports, Median (IQR)
Healer status	Traditional healers	17	36 (28–89)	41 (31–106)
	Other community members	243	28 (23–34)	32 (27–39)
Gender	Women	85	27 (21–32)	31 (27–37)
	Men	175	29 (24–35)	33 (28.5–40)
Education	Incomplete secondary	5	29 (5–31)	32 (9–36)
	Secondary	183	28 (23–34)	34 (29–40)
	Higher	72	26.5 (22–33)	30.5 (26.75–37.25)
Ecological zone	Foothill and low-mountain zone	7	33 (27–34.5)	39 (31.5–39)
	Lower mountain forest belt	138	28 (23–34)	33 (28–39)
	Middle mountain forest belt	37	28 (24–32)	31 (29–36)
	Upper mountain forest and forest–meadow transition	40	28.5 (23.75–36.25)	34.5 (28.5–41)
	High-mountain subalpine meadows	19	29 (22.5–34)	33 (26–39)
	High-mountain alpine meadows and rocky habitats	19	28 (21–33.5)	32 (27.5–40)
Ethnolinguistic affiliation	Azerbaijani	167	28 (23–34)	33 (28–39)
	Lezgi	23	32 (25.5–35.5)	36 (28–40.5)
	Tat	20	32 (26.75–38.25)	36 (29–40.25)
	Khinalug	19	28 (21–33.5)	32 (27.5–40)
	Mountain Jewish	15	27 (22–31)	31 (27–36.5)
	Kryts	9	23 (21–31)	27 (26–36)
	Jek	7	25 (19–32.5)	31 (22–39.5)

**Note:** Values are presented as medians and interquartile ranges (IQRs). These results describe only the surveyed informants. Owing to purposive and snowball sampling and unequal group sizes, they should not be interpreted as population-level estimates.

**Table 3:** Distribution of recorded medicinal plant species and use-reports by botanical family (*N* = 193 species, 59 families).

Family	Number of Plant Species	Total Use-Reports (UR)
Amaranthaceae	1	74
Amaryllidaceae	5	212
Anacardiaceae	2	119
Apiaceae	10	466
Asphodelaceae	1	40
Asteraceae	19	1039
Berberidaceae	1	46
Betulaceae	5	174
Boraginaceae	2	48
Brassicaceae	4	230
Campanulaceae	3	61
Cannabaceae	1	57
Capparaceae	1	33
Caprifoliaceae	6	233
Chenopodiaceae	3	110
Colchicaceae	2	29
Cornaceae	1	54
Cucurbitaceae	3	94
Cupressaceae	1	17
Elaeagnaceae	2	163
Equisetaceae	1	76
Fabaceae	13	429
Fagaceae	2	80
Gentianaceae	3	102
Geraniaceae	2	91
Hypericaceae	2	154
Juglandaceae	1	65
Lamiaceae	22	1303
Linaceae	1	17
Lythraceae	1	75
Malvaceae	5	289
Moraceae	2	149
Nitrariaceae	1	42
Oleaceae	2	29
Onagraceae	1	22
Orchidaceae	3	120
Papaveraceae	3	131
Pedaliaceae	1	60
Plantaginaceae	5	226
Polygalaceae	2	52
Polygonaceae	3	136
Portulacaceae	1	27
Primulaceae	1	34
Ranunculaceae	3	74
Rhamnaceae	2	54
Rosaceae	18	922
Rubiaceae	2	65
Rutaceae	1	21
Salicaceae	1	42
Santalaceae	1	13
Sapindaceae	1	77
Scrophulariaceae	2	61
Solanaceae	3	52
Taxaceae	1	23
Thymelaeaceae	1	17
Verbenaceae	1	31
Viburnaceae	2	79
Violaceae	2	77
Urticaceae	1	66

**Table 4:** Medicinal plants used in traditional medicine in the Guba District, including voucher codes, vernacular names, plant parts used, preparation methods, routes of administration, reported uses, and quantitative ethnobotanical indices (*n* = 260). **Source note:** All vernacular plant names presented in the table were recorded directly from informants during the field interviews. Their spelling and linguistic attribution were subsequently cross-checked against comparative lexical materials from the Intercontinental Dictionary Series (IDS) for Budukh, Khinalug, Kryts, Judeo-Tat, and the Guba dialect of Lezgian, M. Babakhanov’s list of Lezgian phytonyms, and published sources on Juhuri plant names. Vernacular forms that could not be reliably matched to the corresponding taxon at the species level were not included. **Language codes:** Vernacular names without a language code are in Azerbaijani. L—Lezgian; T—Tat; X—Khinalug; G—Kryts; B—Budukh; Y—Juhuri (the language of the Mountain Jews).

Scientific Name	Voucher Code	Vernacular Name (s)	Reported Use (Emic Description)	Analytical Disease Category	Informant Citations	*FL* (%)	*FC*	Plant Part(s)	Preparation Method	Route of Administration	*N*	*FUV*	*RFC*	*ΣU*	*UV*
**Family Amaranthaceae**															
*Amaranthus retroflexus* L.	AZE-IJ-001	Pəncər, amarant	To cleanse the blood vessels	Cardiovascular system conditions	25	59.52	42	Herb	Added directly to food	Oral	260	0.28	0.16	74	0.28
			For liver problems	Hepatobiliary conditions	18	42.86					260				
			To help clear phlegm	Respiratory system conditions	31	73.81					260				
**Family Amaryllidaceae**															
*Allium cepa* L.	AZE-IJ-005	Soğan **Çiç’ek^L^** **Cicək^G^**	For the common cold	Respiratory system conditions	34	80.95	42	Bulb	Used fresh	Oral	260	0.16	0.16	60	0.23
			For toothache	Oral and dental conditions	26	61.9									
*Allium oreophilum* C.A.Mey.	AZE-IJ-006	Soğan **Sarimsok^T^; Sir^T^; Sirçui^T^; Sirsıç^T^** **Cincilim^X^** **Jut^G^; Məsaser^G^** **Lamzu^B^; Şanak^B^**	For the common cold	Respiratory system conditions	21	100	21	Bulb	Used fresh	Oral			0.08	21	0.08
*Allium sativum* L.	AZE-IJ-007	Sarımsaq **Serg^L^** **Sir^T^**	To cleanse the blood vessels	Cardiovascular system conditions	47	88.68	53	Bulb	Used fresh	Oral			0.2	66	0.25
			For intestinal worms	Digestive system conditions	19	35.85									
*Allium ursinum* L.	AZE-IJ-008	Xalyar (ayı soğanı) **Ferar^L^; Surar^L^** **Ǯäärä^Y^; Xanku^Y^**	For stomach and intestinal problems	Digestive system conditions	49	100	49	Bulb	Decoction	Oral			0.19	49	0.19
*Galanthus alpinus* Sosn. var. Alpinus	AZE-IJ-009	Xədicəgülü	For stomach and intestinal problems	Digestive system conditions	16	100	16	Bulb	Cooking/boiling	Oral			0.06	16	0.06
**Family Anacardiaceae**															
*Cotinus coggygria* Scop.	AZE-IJ-010	Sarağan	For pain and swelling	Respiratory system conditions	54	100	54	Leaf	Decoction	Oral	260	0.23	0.21	54	0.21
*Rhus coriaria* L.	AZE-IJ-011	Sumaq	For stomach and intestinal problems	Digestive system conditions	46	75.41	61	Fruit	Decoction	Oral			0.23	65	0.25
			For pain and swelling	Respiratory system conditions	19	31.15									
**Family Apiaceae**															
*Anethum graveolens* L.	AZE-IJ-012	Şüyüd **Şivit^L^** **Morux-šiviti^Y^** **Şivit^G^**	For high blood pressure	Cardiovascular system conditions	22	28.57	77	Flower	Decoction	Oral	260	0.18	0.3	100	0.38
			To improve appetite	Digestive system conditions	15	19.48									
			For breathing difficulties	Respiratory system conditions	29	37.66									
			To cleanse the blood vessels	Cardiovascular system conditions	34	44.16									
*Bupleurum wittmannii* Steven	AZE-IJ-013	Tütəkotu	For skin problems	Dermatological conditions	13	100	13	Herb	Ash application	Topical			0.05	13	0.05
*Carum caucasicum* (M.Bieb.) Boiss.	AZE-IJ-014	Zirə **İferar^L^** **Kujur^X^** **Kurah^G^** **Kurah^B^**	For kidney problems	Genitourinary system conditions	14	36.84	38	Seed	Cooking/boiling	Oral			0.15	53	0.2
			To help clear phlegm	Respiratory system conditions	30	78.95									
			For bedwetting	Genitourinary system conditions	9	23.68									
*Coriandrum sativum* L.	AZE-IJ-015	Keşniş **K’eşniş^L^**	To promote bile flow	Hepatobiliary conditions	27	72.97	37	Fruit	Added directly to food	Oral			0.14	45	0.17
			For headache	Nervous system and sleep-related conditions	18	48.65									
*Ferula caspica* M.Bieb.	AZE-IJ-016	İlankölgəsi	For skin problems	Dermatological conditions	14	100	14	Root	Cooking/boiling	Oral			0.05	14	0.05
*Foeniculum vulgare* Mill.	AZE-IJ-017	Razyana	For abdominal bloating and gas	Digestive system conditions	38	52.78	72	Fruit	Decoction	Oral			0.28	96	0.37
			For cough	Respiratory system conditions	32	44.44									
			For breathing difficulties	Respiratory system conditions	26	36.11									
*Heracleum sosnowskyi* Manden.	AZE-IJ-018	Baldırğan **Girav^L^; Kudaq’aç’^L^** **Baldarğan^T^; Caldarğan^T^; Candaloş^T^; Gandaloş^T^; Xarapenç^T^** **Zig^G^**	For skin problems	Dermatological conditions	30	100	30	Fruit	Fruit juice	Oral			0.12	30	0.12
*Pimpinella anisum* L.	AZE-IJ-019	Cirə **Tuntuma^T^**	For mouth sores	Oral and dental conditions	17	39.53	43	Fruit	Inhalation	Inhalation			0.17	53	0.2
			For cough	Respiratory system conditions	36	83.72									
*Prangos ferulacea* Lindl.	AZE-IJ-020	Çaşır **İlançolğasi^T^**	For inflamed gums	Oral and dental conditions	21	67.74	31	Herb	Used fresh	Oral			0.12	40	0.15
			For swollen gums	Oral and dental conditions	19	61.29									
*Zosima absinthiifolia* (Vent.) Link	AZE-IJ-021	Atıl-batıl	For women’s health problems	Genitourinary system conditions	15	78.95	19	Herb	Decoction	Oral			0.07	22	0.08
			For hemorrhoids	Digestive system conditions	7	36.84									
**Family Asphodelaceae**															
*Eremurus azerbajdzhanicus* Kharkev.	AZE-IJ-022	Çiriş	For stomach and intestinal problems	Digestive system conditions	40	100	40	Herb	Used fresh	Oral	260	0.15	0.15	40	0.15
**Family Asteraceae**															
*Achillea millefolium* L.	AZE-IJ-023	Boymadərən **Şarçiq’al-ar^L^** **Mərmər^Y^**	For skin problems	Dermatological conditions	17	47.22	36	Herb	Aqueous/alcoholic extract	Topical	260	0.21	0.14	56	0.22
			For hair loss	Dermatological conditions	20	55.56									
			For pain and swelling	Respiratory system conditions	19	52.78									
*Anthemis cotula* L.	AZE-IJ-024	Cığır gözü	For headache	Nervous system and sleep-related conditions	22	57.89	38	Flower	Decoction	Oral			0.15	49	0.19
			For healing wounds	Dermatological conditions	10	26.32									
			For toothache	Oral and dental conditions	17	44.74									
*Arctium lappa* L.	AZE-IJ-025	Atpıtrağı **Parq’ul^L^** **Palpatu^G^** **Pehək^B^; Pek^B^**	For diabetes	Endocrine and metabolic conditions	25	39.06	64	Root	Cooking/boiling	Oral			0.25	77	0.3
			For hair loss	Dermatological conditions	52	81.25									
*Artemisia absinthium* L.	AZE-IJ-026	Yovşan **Yavşan^L^; Tüq’ülq’al^L^; Req’eq’ül^L^**	For skin problems	Dermatological conditions	16	39.02	41	Herb	Aqueous/alcoholic extract	Topical			0.16	56	0.22
			To improve appetite	Digestive system conditions	29	70.73									
			To calm the nerves	Nervous system and sleep-related conditions	11	26.83									
*Bidens tripartita* L.	AZE-IJ-027	Yatıqqanqal **Ts’ehrekarç^L^**	For skin problems	Dermatological conditions	54	83.08	65	Herb	Poultice	Topical			0.25	80	0.31
			For swollen veins in the legs	Cardiovascular system conditions	26	40									
*Calendula arvensis* L.	AZE-IJ-028	Gülümbahar	For skin problems	Dermatological conditions	22	36.67	60	Flower	Aqueous/alcoholic extract	Topical			0.23	79	0.3
			For kidney problems	Genitourinary system conditions	37	61.67									
			For swollen gums	Oral and dental conditions	20	33.33									
*Centaurea phrygia* subsp. *salicifolia* (M.Bieb. ex Willd.) Mikheev	AZE-IJ-029	Güləvər	For fever	Respiratory system conditions	9	33.33	27	Flower	Decoction	Oral			0.1	32	0.12
			For difficulty sleeping	Nervous system and sleep-related conditions	23	85.19									
*Cichorium intybus* L.	AZE-IJ-030	Kasnı **K’alam-ar^L^; Qirxdügüm^L^; Nadriş^L^**	For stomach and intestinal problems	Digestive system conditions	20	44.44	45	Root	Cooking/boiling	Oral			0.17	61	0.23
			For breathing difficulties	Respiratory system conditions	23	51.11									
			For swollen gums	Oral and dental conditions	18	40									
*Helichrysum rubicundum* (K.Koch) Bornm.	AZE-IJ-031	Ödotu **Qulax^L^; Beyşütq’ver^L^**	For liver problems	Hepatobiliary conditions	19	38.78	49	Flower	Decoction	Oral			0.19	62	0.24
			To promote bile flow	Hepatobiliary conditions	26	53.06									
			To cleanse the blood vessels	Cardiovascular system conditions	17	34.69									
*Inula helenium* L.	AZE-IJ-032	Andız **Anduz^L^**	For diabetes	Endocrine and metabolic conditions	29	35.8	81	Rhizome	Cooking/boiling	Oral			0.31	101	0.39
			To help clear phlegm	Respiratory system conditions	44	54.32									
			For kidney problems	Genitourinary system conditions	28	34.57									
*Lactuca tatarica* (L.) C.A.Mey.	AZE-IJ-033	Kahı **Kahu^L^**	To improve appetite	Digestive system conditions	14	100	14	Leaf	Added directly to food	Oral			0.05	14	0.05
*Matricaria aurea* (Loefl.) Sch.Bip.	AZE-IJ-034	Mollabaşı	For pain and swelling	Respiratory system conditions	20	45.45	44	Flower	Decoction	Oral			0.17	53	0.2
			For hemorrhoids	Digestive system conditions	33	75									
*Pseudopodospermum szowitzii* (DC.) Kuth.	AZE-IJ-035	Keçiyemliyi	For skin problems	Dermatological conditions	17	100	17	Root	Cooking/boiling	Oral			0.07	17	0.07
*Silybum marianum* (L.) Gaertn.	AZE-IJ-036	Alaqanqal **Şelal^T^**	For liver problems	Hepatobiliary conditions	42	84	50	Seed	Poultice	Topical			0.19	58	0.22
			For skin problems	Dermatological conditions	16	32									
*Solidago virgaurea* L.	AZE-IJ-037	Qızılsəbət	For stomach and intestinal problems	Digestive system conditions	22	68.75	32	Herb	Decoction	Oral			0.12	37	0.14
			For kidney problems	Genitourinary system conditions	15	46.88									
*Tanacetum coccineum* (Willd.) Grierson	AZE-IJ-038	Dağtərxunu	For stomach and intestinal problems	Digestive system conditions	34	82.93	41	Flower	Decoction	Oral			0.16	51	0.2
			For swollen veins in the legs	Cardiovascular system conditions	17	41.46									
*Taraxacum sonchoides* (D.Don) Sch.Bip.	AZE-IJ-039	Zəncirotu **Nek’edqhaç^L^; Nekqhaç^L^; Psis-ar^L^** **Yamlik^Y^**	To cleanse the blood vessels	Cardiovascular system conditions	22	38.6	57	Root	Cooking/boiling	Oral			0.22	67	0.26
			For liver problems	Hepatobiliary conditions	18	31.58									
			For stomach and intestinal problems	Digestive system conditions	27	47.37									
*Tragopogon tuberosus* K.Koch.	AZE-IJ-040	Yemlik **Şing^T^** **Alemink^X^** **Mhunkai^G^; Yohahun^G^**	For high blood pressure	Cardiovascular system conditions	11	100	11	Leaf	Decoction	Oral			0.04	11	0.04
*Tussilago farfara* L.	AZE-IJ-041	Dəvədabanı **T’engir^L^** **Çurki^G^; Çurti^G^; Dolmajapagə^G^; Tolpaka^G^**	To help clear phlegm	Respiratory system conditions	42	61.76	68	Leaf	Decoction	Oral			0.26	78	0.3
			For cough	Respiratory system conditions	36	52.94									
**Family Berberidaceae**															
*Berberis vulgaris* L.	AZE-IJ-042	Zirinc **Mert-yer^L^** **Sum^T^; Ziriş^T^; Zirişk^T^** **Mirape^X^; Siriş^X^** **Siriş^G^**	To promote bile flow	Hepatobiliary conditions	46	100	46	Fruit	Decoction	Oral	260	0.18	0.18	46	0.18
**Family Betulaceae**															
*Alnus glutinosa* subsp. *barbata* (C.A.Mey.) Yalt.	AZE-IJ-043	Qızılağac **Maq^L^**	For stomach and intestinal problems	Digestive system conditions	47	100	47	Fruit	Decoction	Oral	260	0.13	0.18	47	0.18
*Alnus incana* (L.) Moench	AZE-IJ-044	Qızılağac **Maq^L^**	For stomach and intestinal problems	Digestive system conditions	33	100	33	Fruit	Decoction	Oral	260		0.13	33	0.13
*Betula pendula* Roth	AZE-IJ-045	Tozağacı **Wer$\overset{⌵}{x}$in tar^L^** **Werx^X^** **Merx^G^** **Merx^B^** **Bereza^Y^** **Verxh^L^**	To increase urination	Genitourinary system conditions	27	100	27	Leaf	Decoction	Oral	260		0.1	27	0.1
*Carpinus orientalis* Mill.	AZE-IJ-046	Vələs	For skin problems	Dermatological conditions	28	100	28	Leaf	Poultice	Topical	260		0.11	28	0.11
*Corylus avellana* L.	AZE-IJ-047	Fındıq **Şümyağ^L^**	For kidney problems	Genitourinary system conditions	23	74.19	31	Fruit	Added directly to food	Oral	260		0.12	39	0.15
			For headache	Nervous system and sleep-related conditions	16	51.61									
**Family Boraginaceae**															
*Anchusa azurea* Mill.	AZE-IJ-048	Sümsürgən	For women’s health problems	Genitourinary system conditions	30	100	30	Herb	Decoction	Oral	260	0.09	0.12	30	0.12
*Symphytum caucasicum* M.Bieb.	AZE-IJ-049	Xəndəkotu	For skin problems	Dermatological conditions	18	100	18	Herb	Aqueous/alcoholic extract	Topical	260		0.07	18	0.07
**Family Brassicaceae**															
*Brassica oleracea* L.	AZE-IJ-050	Kələm **Kelem^L^** **Kalam^G^**	For stomach ulcers	Digestive system conditions	39	81.25	48	Leaf	Fruit juice	Oral	260	0.22	0.18	70	0.27
			For hemorrhoids	Digestive system conditions	9	18.75									
			For skin problems	Dermatological conditions	22	45.83									
*Capsella bursa-pastoris* Medik.	AZE-IJ-051	Quşəppəyi **Temezxan^L^** **Gagulaguşin^T^; Kogolla^T^; Pilpilaç^T^** **Quşapəyi^X^** **Quşapəyi^G^; Tere^G^**	For kidney problems	Genitourinary system conditions	20	35.71	56	Herb	Used fresh	Oral	260		0.22	69	0.27
			For women’s health problems	Genitourinary system conditions	32	57.14									
			For joint and rheumatic pain	Musculoskeletal conditions	17	30.36									
*Mutarda arvensis* (L.) D.A.German	AZE-IJ-052	Xardal	For the common cold	Respiratory system conditions	19	100	19	Seed	Inhalation	Inhalation	260		0.07	19	0.07
*Raphanus raphanistrum* subsp. *sativus* (L.) Domin	AZE-IJ-053	Turp **Turp^L^; Ç’ulav turp^L^**	For cough	Respiratory system conditions	30	49.18	61	Tuber	Added directly to food	Oral	260		0.23	72	0.28
			To help clear phlegm	Respiratory system conditions	42	68.85									
**Family Campanulaceae**															
*Asyneuma campanuloides* (M.Bieb.) Bornm.	AZE-IJ-054	Zəngçiçəyi **Kurkur-ar^L^**	For joint and rheumatic pain	Musculoskeletal conditions	12	44.44	27	Herb	Decoction	Oral	260	0.08	0.1	31	0.12
			For women’s health problems	Genitourinary system conditions	19	70.37									
*Campanula glomerata* L.	AZE-IJ-055	Zəngçiçəyi **Kurkur-ar^L^**	For pain and swelling	Respiratory system conditions	22	100	22	Herb	Aqueous/alcoholic extract	Oral	260		0.08	22	0.08
*Campanula sarmatica* Ker Gawl.	AZE-IJ-056	Zəngçiçəyi **Kurkur-ar^L^**	For skin problems	Dermatological conditions	8	100	8	Herb	Massage	Topical	260		0.03	8	0.03
**Family Cannabaceae**															
*Humulus lupulus* L.	AZE-IJ-057	Mayasarmaşığı **Tereful-ar^L^; Juvan-ar^L^**	To calm the nerves	Nervous system and sleep-related conditions	41	83.67	49	Flower	Decoction	Oral	260	0.22	0.19	57	0.22
			For difficulty sleeping	Nervous system and sleep-related conditions	16	32.65									
**Family Capparaceae**															
*Capparis spinosa* var. *herbacea* (Willd.) Fici	AZE-IJ-058	Kövər **Peqhrexali^L^; Qaqats’^L^; Kever^L^**	For stomach and intestinal problems	Digestive system conditions	33	100	33	Fruit	Fruit juice	Oral	260	0.13	0.13	33	0.13
**Family Caprifoliaceae**															
*Cephalaria gigantea* (Ledeb.) Bobrov	AZE-IJ-059	Qantəpər	For the common cold	Respiratory system conditions	55	100	55	Flower	Inhalation	Inhalation	260	0.15	0.21	55	0.21
*Dipsacus laciniatus* L.	AZE-IJ-060	Fırçaotu	For women’s health problems	Genitourinary system conditions	42	100	42	Flower	Decoction	Oral	260		0.16	42	0.16
*Lomelosia caucasica* (M.Bieb.) Greuter & Burdet	AZE-IJ-061	Skabioza	For diabetes	Endocrine and metabolic conditions	26	76.47	34	Flower	Decoction	Oral	260		0.13	39	0.15
			For high blood pressure	Cardiovascular system conditions	13	38.24									
*Lonicera iberica* M.Bieb.	AZE-IJ-062	Doqquzdon	To improve appetite	Digestive system conditions	21	100	21	Fruit	Decoction	Oral	260		0.08	21	0.08
*Valeriana alliariifolia* Vahl	AZE-IJ-063	Pişikotu	To calm the nerves	Nervous system and sleep-related conditions	16	51.61	31	Rhizome	Cooking/boiling	Oral	260		0.12	45	0.17
			For difficulty sleeping	Nervous system and sleep-related conditions	29	93.55									
*Valeriana alliariifolia var. tiliifolia* (Troickij) V.E.Avet.	AZE-IJ-064	Pişikotu	To calm the nerves	Nervous system and sleep-related conditions	11	44	25	Rhizome	Cooking/boiling	Oral	260		0.1	31	0.12
			For difficulty sleeping	Nervous system and sleep-related conditions	20	80									
**Family Chenopodiaceae**															
*Beta vulgaris* L.	AZE-IJ-002	Çuğundur	To cleanse the blood vessels	Cardiovascular system conditions	16	45.71	35	Tuber	Added directly to food	Oral	260	0.14	0.13	58	0.22
			For diabetes	Endocrine and metabolic conditions	22	62.86					260				
			For liver problems	Hepatobiliary conditions	20	57.14					260				
*Blitum virgatum* L.	AZE-IJ-003	Tərə	For pain and swelling	Respiratory system conditions	15	100	15	Herb	Fruit juice	Oral	260		0.06	15	0.06
											260				
*Spinacia oleracea* L.	AZE-IJ-004	İspanaq	For constipation	Digestive system conditions	37	100	37	Leaf	Used fresh	Oral	260		0.14	37	0.14
											260				
**Family Colchicaceae**															
*Colchicum speciosum* Steven	AZE-IJ-065	İtboğan	For skin problems	Dermatological conditions	12	100	12	Tuber	Aqueous/alcoholic extract	Topical	260	0.06	0.05	12	0.05
*Colchicum trigynum* (Steven ex Adams) *Stearn*	AZE-IJ-066	Danaqıran	For pain and swelling	Respiratory system conditions	17	100	17	Tuber	Decoction	Oral	260		0.07	17	0.07
**Family Cornaceae**															
*Cornus mas* L.	AZE-IJ-067	Zoğal **Soğal^X^** **Kizil^B^** **Cimel^G^**	For stomach and intestinal problems	Digestive system conditions	54	100	54	Fruit	Added directly to food	Oral	260	0.21	0.21	54	0.21
**Family Cucurbitaceae**															
*Bryonia aspera* Steven ex Fisch.	AZE-IJ-068	Küstüşam	For skin problems	Dermatological conditions	16	100	16	Leaf	Aqueous/alcoholic extract	Topical	260	0.12	0.06	16	0.06
*Ecballium elaterium* (L.) A.Rich.	AZE-IJ-069	İtxiyarı	For pain and swelling	Respiratory system conditions	14	100	14	Fruit	Fruit juice	Oral	260		0.05	14	0.05
*Cucurbita pepo* L.	AZE-IJ-070	Balqabaq, boranı **Buran^L^**, **$\bar{Q}$abaɣ^X^** **Qʼabaɣ^G^; Qʼäbäɣ^G^ $\bar{Q}$abaɣ^B^** **Küdü^Y^** **Qəbəx^G^**	For liver problems	Hepatobiliary conditions	29	60.42	48	Fruit	Added directly to food	Oral	260		0.18	64	0.25
			To improve appetite	Digestive system conditions	22	45.83									
			For intestinal worms	Digestive system conditions	13	27.08									
**Family Cupressaceae**															
*Juniperus communis* var. *saxatilis* Pall.	AZE-IJ-071	Ardıc **Riperag^L^; Muk’rag^L^; Kupultsaz^L^**	To increase urination	Genitourinary system conditions	17	100	17	Fruit	Decoction	Oral	260	0.07	0.07	17	0.07
**Family Elaeagnaceae**															
*Elaeagnus angustifolia* L.	AZE-IJ-072	İydə **İgidtar^L^; Xhütük^L^**	For pain and swelling	Respiratory system conditions	19	61.29	31	Fruit	Used fresh	Oral	260	0.31	0.12	39	0.15
			For mouth sores	Oral and dental conditions	20	64.52									
*Hippophae rhamnoides* L.	AZE-IJ-073	Çaytikanı **Yigi^L^; Yigidul^L^; İgdi^L^** **Galebizım^T^; Karsum^T^** **Aktulak^G^** **Kilakoholo^B^; Kotkolo^B^**	For healing wounds	Dermatological conditions	48	60.76	79	Fruit	Used fresh	Oral	260		0.3	124	0.48
			For skin problems	Dermatological conditions	34	43.04									
			For stomach ulcers	Digestive system conditions	42	53.16									
**Family Equisetaceae**															
*Equisetum arvense* L.	AZE-IJ-074	Qatırquyruğu **Vak’an kanab^L^**	To increase urination	Genitourinary system conditions	33	49.25	67	Herb	Decoction	Oral	260	0.29	0.26	76	0.29
			For kidney problems	Genitourinary system conditions	28	41.79									
			To help pass kidney stones	Genitourinary system conditions	15	22.39									
**Family Fabaceae**															
*Astragalus caucasicus* Pall.	AZE-IJ-075	Gəvən **Majv^L^; Maçvar^L^**	For skin problems	Dermatological conditions	37	78.72	47	Herb	Fruit juice	Oral	260	0.13	0.18	51	0.2
			For swollen veins in the legs	Cardiovascular system conditions	14	29.79									
*Galega orientalis* Lam.	AZE-IJ-076	Çəpişotu	For diabetes	Endocrine and metabolic conditions	25	100	25	Herb	Decoction	Oral	260		0.1	25	0.1
*Glycyrrhiza glabra* L.	AZE-IJ-077	Biyan **Biyan^L^; İk’i^L^**	For cough	Respiratory system conditions	56	66.67	84	Root	Cooking/boiling	Oral	260		0.32	118	0.45
			For pain and swelling	Respiratory system conditions	25	29.76									
			For breathing difficulties	Respiratory system conditions	37	44.05									
*Lathyrus hirsutus* L.	AZE-IJ-078	Gülülcə	For diabetes	Endocrine and metabolic conditions	14	100	14	Herb	Decoction	Oral	260		0.05	14	0.05
*Medicago* × varia *Martyn*	AZE-IJ-079	Qarayonca **Qunj^L^; Spax^L^**	For skin problems	Dermatological conditions	30	100	30	Herb	Poultice	Topical	260		0.12	30	0.12
*Medicago minima* (L.) Bartal.	AZE-IJ-080	Qarayonca **Qunj^L^; Spax^L^**	For healing wounds	Dermatological conditions	20	100	20	Herb	Decoction	Oral	260		0.08	20	0.08
*Melilotus officinalis* (L.) Lam.	AZE-IJ-081	Xəşəmbül **Taq-ar^L^; Takun-ar^L^; Tsulu-yar^L^**	For healing wounds	Dermatological conditions	19	100	19	Herb	Decoction	Oral	260		0.07	19	0.07
*Onobrychis petraea* (M.Bieb. ex Willd.) Fisch.	AZE-IJ-082	Xaşa	For diabetes	Endocrine and metabolic conditions	25	100	25	Herb	Decoction	Oral	260		0.1	25	0.1
*Onobrychis radiata* (Desf.) M.Bieb.	AZE-IJ-083	Xaşa	For pain and swelling	Respiratory system conditions	17	100	17	Herb	Decoction	Oral	260		0.07	17	0.07
*Ononis arvensis* L.	AZE-IJ-084	Paxlakolu	To increase urination	Genitourinary system conditions	23	92	25	Root	Cooking/boiling	Oral	260		0.1	29	0.11
			For kidney problems	Genitourinary system conditions	6	24									
*Trifolium ochroleucon* subsp. *ochroleucon*	AZE-IJ-085	Üçyarpaq yonca **İnji^L^; Pudraq’ey^L^** **Çəçəlezote^T^**	For pain and swelling	Respiratory system conditions	25	73.53	34	Herb	Decoction	Oral	260		0.13	42	0.16
			For women’s health problems	Genitourinary system conditions	17	50									
*Trigonella spicata* Sm.	AZE-IJ-086	Şəmbələ	For healing wounds	Dermatological conditions	31	100	31	Herb	Decoction	Oral	260		0.12	31	0.12
*Vicia lathyroides* L.	AZE-IJ-087	Lərgə **Q’ifrexar-ar^L^**	For women’s health problems	Genitourinary system conditions	8	100	8	Herb	Decoction	Oral	260		0.03	8	0.03
**Family Fagaceae**															
*Fagus orientalis* Lipsky	AZE-IJ-088	Fısdıq **Pipin tar^L^** **Pip^X^; Fɨs$\bar{t}$ɨ$\bar{q}$^X^** **Pip^G^** **Pip^B^** **Činor^Y^**	For healing wounds	Dermatological conditions	33	100	33	Fruit	Decoction	Oral	260	0.15	0.13	33	0.13
*Quercus castaneifolia* C.A.Mey.	AZE-IJ-089	Palıd **Meɣün tar^L^** **Palɨd^X^** **Meɣ^G^; Palut^G^** **Palɨt^B^** **Paluddor^Y^**	For mouth sores	Oral and dental conditions	27	65.85	41	Bark	Cooking/boiling	Oral	260		0.16	47	0.18
			For toothache	Oral and dental conditions	20	48.78									
**Family Gentianaceae**															
*Centaurium erythraea* Rafn	AZE-IJ-090	Qızılçətir, isitməotu **Şemdegan-ar^L^**	For bedwetting	Genitourinary system conditions	10	19.23	52	Herb	Decoction	Oral	260	0.13	0.2	60	0.23
			For joint and rheumatic pain	Musculoskeletal conditions	16	30.77									
			To improve appetite	Digestive system conditions	11	21.15									
			For hemorrhoids	Digestive system conditions	23	44.23									
*Gentiana cruciata L.*	AZE-IJ-091	Acıçiçək	For stomach and intestinal problems	Digestive system conditions	25	100	25	Herb	Decoction	Oral	260		0.1	25	0.1
*Gentiana septemfida* Pall.	AZE-IJ-092	Acıçiçək	To promote bile flow	Hepatobiliary conditions	17	100	17	Herb	Decoction	Oral	260		0.07	17	0.07
**Family Geraniaceae**															
*Geranium columbinum* L.	AZE-IJ-093	Ətirşah	For mouth sores	Oral and dental conditions	36	83.72	43	Herb	Inhalation	Inhalation	260	0.18	0.17	48	0.18
			To help pass kidney stones	Genitourinary system conditions	12	27.91									
*Geranium sibiricum* L.	AZE-IJ-094	Ətirşah	For irregular heartbeat	Cardiovascular system conditions	23	71.88	32	Herb	Decoction	Oral	260		0.12	43	0.17
			For hemorrhoids	Digestive system conditions	20	62.5									
**Family Hypericaceae**															
*Hypericum linarioides* Bosse	AZE-IJ-095	Boyçiçəyi **Helbe-yar^L^; Hiliba-yar^L^; Vişsebeb^L^**	For difficulty sleeping	Nervous system and sleep-related conditions	32	74.42	43	Herb	Decoction	Oral	260	0.3	0.17	59	0.23
			To calm the nerves	Nervous system and sleep-related conditions	27	62.79									
*Hypericum perforatum* L.	AZE-IJ-096	Adi dazı **Helbe-yar^L^; Hiliba-yar^L^; Vişsebeb^L^** **Şimşıt^T^**	For healing wounds	Dermatological conditions	25	33.78	74	Herb	Decoction	Oral	260		0.28	95	0.37
			For diabetes	Endocrine and metabolic conditions	22	29.73									
			For difficulty sleeping	Nervous system and sleep-related conditions	17	22.97									
			For pain and swelling	Respiratory system conditions	31	41.89									
**Family Juglandaceae**															
*Juglans regia* L.	AZE-IJ-097	Qoz **K’erets^L^; Qharqhutar^L^** **Əbirt^G^**	For heart and circulatory problems	Cardiovascular system conditions	39	75	52	Fruit	Used fresh	Oral	260	0.25	0.2	65	0.25
			To cleanse the blood vessels	Cardiovascular system conditions	26	50									
**Family Lamiaceae**															
*Ajuga orientalis* L.	AZE-IJ-098	Sığırdili	For healing wounds	Dermatological conditions	37	100	37	Herb	Decoction	Oral	260	0.23	0.14	37	0.14
*Betonica officinalis* L.	AZE-IJ-099	Nəmgül	For women’s health problems	Genitourinary system conditions	29	100	29	Herb	Decoction	Oral	260		0.11	29	0.11
*Dracocephalum botryoides* Steven	AZE-IJ-100	İlanbaşı	For kidney problems	Genitourinary system conditions	16	64	25	Herb	Decoction	Oral	260		0.1	27	0.1
			To calm the nerves	Nervous system and sleep-related conditions	11	44									
*Lamium album* L.	AZE-IJ-101	Dalamaz	For pain and swelling	Respiratory system conditions	24	38.71	62	Herb	Decoction	Oral	260		0.24	72	0.28
			For kidney problems	Genitourinary system conditions	28	45.16									
			For irregular heartbeat	Cardiovascular system conditions	20	32.26									
*Leonurus quinquelobatus* Gilib.	AZE-IJ-102	Şirquyruğu, damotu **Yavaq’an^L^**	For difficulty sleeping	Nervous system and sleep-related conditions	20	50	40	Herb	Decoction	Oral	260		0.15	47	0.18
			To calm the nerves	Nervous system and sleep-related conditions	27	67.5									
*Marrubium vulgare* L.	AZE-IJ-103	İtotu	For skin problems	Dermatological conditions	15	71.43	21	Herb	Aqueous/alcoholic extract	Topical	260		0.08	28	0.11
			To help pass kidney stones	Genitourinary system conditions	13	61.9									
*Melissa officinalis* L.	AZE-IJ-104	Bədrənc, limonlu nanə, balotu	For irregular heartbeat	Cardiovascular system conditions	25	39.06	64	Herb	Decoction	Oral	260		0.25	71	0.27
			To calm the nerves	Nervous system and sleep-related conditions	20	31.25									
			For heart and circulatory problems	Cardiovascular system conditions	26	40.63									
*Mentha aquatica* L.	AZE-IJ-105	Yarpız **Purni^L^; Çidriq’^L^; Sp’ra^L^; Şanat^L^** **Naano^Y^** **Nanü^T^** **Laha^X^** **Çurki^G^; Çurti^G^; Kaç^G^** **Çurtha^B^** **Cərdəh^G^**	For difficulty sleeping	Nervous system and sleep-related conditions	43	44.33	97	Herb	Decoction	Oral	260		0.37	136	0.52
			To help pass kidney stones	Genitourinary system conditions	16	16.49									
			To calm the nerves	Nervous system and sleep-related conditions	40	41.24									
			For headache	Nervous system and sleep-related conditions	37	38.14									
*Mentha longifolia* (L.) Huds.	AZE-IJ-106	Uzunyarpaq nanə **Purni^L^; Çidriq’^L^; Sp’ra^L^; Şanat^L^** **Naano^Y^** **Purinat^T^** **Laha^X^** **Çurki^G^; Çurti^G^; Kaç^G^** **Çurtha^B^**	For skin problems	Dermatological conditions	18	24.66	73	Leaf	Decoction	Oral	260		0.28	90	0.35
			For abdominal bloating and gas	Digestive system conditions	33	45.21									
			For headache	Nervous system and sleep-related conditions	28	38.36									
			For irregular heartbeat	Cardiovascular system conditions	11	15.07									
*Nepeta betonicifolia* C.A.Mey.	AZE-IJ-107	Pişiknanəsi	For women’s health problems	Genitourinary system conditions	24	39.34	61	Leaf	Decoction	Oral	260		0.23	70	0.27
			For pain and swelling	Respiratory system conditions	27	44.26									
			For kidney problems	Genitourinary system conditions	19	31.15									
*Origanum vulgare* L.	AZE-IJ-108	Qaraqınıq **Qaraçip^L^; Ç’ulavq’al^L^** **Purinə^Y^; Qara purinə^Y^**	For hair loss	Dermatological conditions	33	42.31	78	Herb	Fruit juice	Oral	260		0.3	128	0.49
			To calm the nerves	Nervous system and sleep-related conditions	38	48.72									
			For breathing difficulties	Respiratory system conditions	57	73.08									
*Phlomis herba-venti* subsp. *pungens* (Willd.) Maire ex DeFilipps	AZE-IJ-109	Odotu	For kidney problems	Genitourinary system conditions	29	100	29	Herb	Decoction	Oral	260		0.11	29	0.11
*Prunella vulgaris* L.	AZE-IJ-110	Boğazotu	For pain and swelling	Respiratory system conditions	43	100	43	Herb	Decoction	Oral	260		0.17	43	0.17
*Salvia aethiopis* L.	AZE-IJ-111	Sürvə	For skin problems	Dermatological conditions	31	100	31	Leaf	Massage	Topical	260		0.12	31	0.12
*Salvia glutinosa* L.	AZE-IJ-112	Sürvə	For healing wounds	Dermatological conditions	10	13.89	72	Leaf	Decoction	Oral; Gargle/Mouth rinse	260		0.28	79	0.3
			For joint and rheumatic pain	Musculoskeletal conditions	18	25									
			For women’s health problems	Genitourinary system conditions	22	30.56									
			For swollen gums	Oral and dental conditions	20	27.78									
			For hemorrhoids	Digestive system conditions	9	12.5									
*Satureja hortensis* L.	AZE-IJ-113	Çöl nanəsi	For stomach and intestinal problems	Digestive system conditions	14	20.9	67	Herb	Decoction	Oral	260		0.26	74	0.28
			To increase urination	Genitourinary system conditions	17	25.37									
			For kidney problems	Genitourinary system conditions	23	34.33									
			For hair loss	Dermatological conditions	20	29.85									
*Scutellaria orientalis* L.	AZE-IJ-114	Başlıqotu	For the common cold	Respiratory system conditions	36	100	36	Herb	Decoction	Oral	260		0.14	36	0.14
*Sideritis montana* L.	AZE-IJ-115	Dəmrək	For pain and swelling	Respiratory system conditions	17	53.13	32	Root	Cooking/boiling	Oral	260		0.12	38	0.15
			For hair loss	Dermatological conditions	21	65.63									
*Stachys pauli* Grossh.	AZE-IJ-116	Poruq	For fever	Respiratory system conditions	17	25.76	66	Herb	Decoction	Oral	260		0.25	73	0.28
			For heart and circulatory problems	Cardiovascular system conditions	56	84.85									
*Teucrium chamaedrys* L.	AZE-IJ-117	Məryəmnoxudu	For skin problems	Dermatological conditions	47	100	47	Herb	Aqueous/alcoholic extract	Topical	260		0.18	47	0.18
*Thymus praecox* subsp. *caucasicus* (Willd. ex Ronniger) Jalas	AZE-IJ-118	Kəklikotu, kəkik **Ts’antar-ar^L^; Qvetreq’al-ar^L^** **Kəklik-otu^Y^** **Merzajü^T^; Purinat^T^; Tazai^T^** **Kəliglotu^X^** **Çengal^G^; Engal^G^** **Çengal^B^; Engal^B^; Kəliglotu^B^; Nurs^B^** **Gügülgütü^G^**	To help clear phlegm	Respiratory system conditions	39	82.98	47	Herb	Decoction	Oral	260		0.18	64	0.25
			For abdominal bloating and gas	Digestive system conditions	25	53.19									
*Ziziphora capitata* L.	AZE-IJ-119	Dağ nanəsi	For inflamed gums	Oral and dental conditions	16	35.56	45	Herb	Decoction	Oral	260		0.17	54	0.21
			For swollen veins in the legs	Cardiovascular system conditions	38	84.44									
**Family Linaceae**															
*Linum austriacum* L.	AZE-IJ-120	Zəyərək	For healing wounds	Dermatological conditions	17	100	17	Fruit	Decoction	Oral	260	0.07	0.07	17	0.07
**Family Lythraceae**															
*Punica granatum* L.	AZE-IJ-121	Nar **Anar^L^**	For high blood pressure	Cardiovascular system conditions	41	69.49	59	Fruit	Used fresh	Oral	260	0.29	0.23	75	0.29
			For difficulty sleeping	Nervous system and sleep-related conditions	23	38.98									
			For headache	Nervous system and sleep-related conditions	11	18.64									
**Family Malvaceae**															
*Alcea kusjariensis* (Iljin ex Grossh.) Iljin	AZE-IJ-122	Xətmigül **Yengetsük^L^**	For breathing difficulties	Respiratory system conditions	20	30.3	66	Leaf	Decoction	Oral	260	0.22	0.25	81	0.31
			For constipation	Digestive system conditions	15	22.73									
			For diabetes	Endocrine and metabolic conditions	27	40.91									
			For skin problems	Dermatological conditions	19	28.79									
*Althaea armeniaca* Ten.	AZE-IJ-123	Bəlğəmotu **Dekurkvaç’^L^**	For inflamed gums	Oral and dental conditions	26	50.98	51	Root	Cooking/boiling	Oral	260		0.2	60	0.23
			For stomach ulcers	Digestive system conditions	34	66.67									
*Malva punctata* (All.) Alef.	AZE-IJ-124	Xətmi	To help clear phlegm	Respiratory system conditions	27	100	27	Herb	Decoction	Oral	260		0.1	27	0.1
*Malva sylvestris* L.	AZE-IJ-125	Əməköməci, balba **Kurkvaç’^L^** **Pülpülə^Y^** **Pilpilaç^T^** **Turkaş^X^** **Kostosu^B^**	For inflamed gums	Oral and dental conditions	14	23.33	60	Leaf	Decoction	Oral	260		0.23	72	0.28
			For breathing difficulties	Respiratory system conditions	36	60									
			For stomach ulcers	Digestive system conditions	22	36.67									
*Tilia cordata* Mill.	AZE-IJ-126	Cökə	For fever	Respiratory system conditions	49	100	49	Flower	Decoction	Oral	260		0.19	49	0.19
**Family Moraceae**															
*Ficus carica* L.	AZE-IJ-127	Əncir **İnžil tar^L^** **İnǯir^X^** **İnǯil^G^** **İnǯil^B^** **Enǯil^Y^**	For cough	Respiratory system conditions	26	38.81	67	Fruit	Used fresh	Oral	260	0.29	0.26	74	0.28
			For breathing difficulties	Respiratory system conditions	18	26.87									
			For heart and circulatory problems	Cardiovascular system conditions	30	44.78									
*Morus alba* L.	AZE-IJ-128	Ağ tut **Req’edtar^L^; Req’eşta^L^**	For liver problems	Hepatobiliary conditions	39	54.93	71	Fruit	Used fresh	Oral	260		0.27	75	0.29
			For breathing difficulties	Respiratory system conditions	22	30.99									
			For toothache	Oral and dental conditions	14	19.72									
**Family Nitrariaceae**															
*Peganum harmala* L.	AZE-IJ-129	Üzərlik **Harmula-yar^L^**	For healing wounds	Dermatological conditions	17	53.13	32	Herb	Decoction; burning of dried aerial parts	Topical; Fumigation	260	0.16	0.12	42	0.16
			For difficulty sleeping	Nervous system and sleep-related conditions	25	78.13									
**Family Oleaceae**															
*Fraxinus excelsior* L.	AZE-IJ-130	Göyrüş **Tsutsuntar^L^**	For pain and swelling	Respiratory system conditions	16	100	16	Leaf	Decoction	Oral	260	0.06	0.06	16	0.06
*Chrysojasminum fruticans* (L.) Banfi	AZE-IJ-131	Jasmin	For healing wounds	Dermatological conditions	13	100	13	Leaf	Fruit juice	Oral	260		0.05	13	0.05
**Family Onagraceae**															
*Epilobium hirsutum* L.	AZE-IJ-132	Onaqra	For skin problems	Dermatological conditions	22	100	22	Herb	Ash application	Topical	260	0.08	0.08	22	0.08
**Family Orchidaceae**															
*Anacamptis pyramidalis* (L.) Rich.	AZE-IJ-133	Bağ səhləbi	For cough	Respiratory system conditions	34	100	34	Tuber	Decoction	Oral	260	0.15	0.13	34	0.13
*Ophrys apifera* Huds.	AZE-IJ-134	Qaş səhləbi	To help clear phlegm	Respiratory system conditions	21	67.74	31	Tuber	Decoction	Oral	260		0.12	38	0.15
			For cough	Respiratory system conditions	17	54.84									
*Orchis mascula* (L.) L.	AZE-IJ-135	Erkək səhləb	For stomach ulcers	Digestive system conditions	15	37.5	40	Tuber	Decoction	Oral	260		0.15	48	0.18
			To help clear phlegm	Respiratory system conditions	33	82.5									
**Family Papaveraceae**															
*Chelidonium majus* L.	AZE-IJ-136	Dəmrovotu **Hetretsük^L^; Mat’an^L^**	To promote bile flow	Hepatobiliary conditions	38	86.36	44	Leaf	Fruit juice; used fresh	Oral; Topical	260	0.17	0.17	55	0.21
			For women’s health problems	Genitourinary system conditions	17	38.64									
*Fumaria asepala* Boiss.	AZE-IJ-137	Şahtərə	To promote bile flow	Hepatobiliary conditions	10	43.48	23	Herb	Decoction	Oral	260		0.09	28	0.11
			For diabetes	Endocrine and metabolic conditions	18	78.26									
*Glaucium elegans* Fisch. & C.A.Mey.	AZE-IJ-138	Buynuzlalə	For cough	Respiratory system conditions	40	93.02	43	Herb	Decoction	Oral	260		0.17	48	0.18
			For headache	Nervous system and sleep-related conditions	8	18.6									
**Family Pedaliaceae**															
*Sesamum indicum* L.	AZE-IJ-139	Küncüt	For liver problems	Hepatobiliary conditions	36	75	48	Seed	Added directly to food	Oral	260	0.23	0.18	60	0.23
			To cleanse the blood vessels	Cardiovascular system conditions	24	50									
**Family Plantaginaceae**															
*Linaria vulgaris* Mill.	AZE-IJ-140	Qurdotu	To promote bile flow	Hepatobiliary conditions	21	67.74	31	Herb	Decoction	Oral	260	0.17	0.12	36	0.14
			To increase urination	Genitourinary system conditions	15	48.39									
*Plantago lanceolata* L.	AZE-IJ-141	Bağayarpağı **Xirenpeş^L^**	For stomach ulcers	Digestive system conditions	27	77.14	35	Leaf	Fruit juice	Oral	260		0.13	46	0.18
			To help clear phlegm	Respiratory system conditions	19	54.29									
*Plantago major* L.	AZE-IJ-142	Bağayarpağı **Damardpeş^L^; Jüdğvel^L^** **Balgadunə^Y^** **Bağalbağe^T^**	For stomach ulcers	Digestive system conditions	39	58.21	67	Leaf	Fruit juice	Oral	260		0.26	80	0.31
			For diabetes	Endocrine and metabolic conditions	28	41.79									
			For joint and rheumatic pain	Musculoskeletal conditions	13	19.4									
*Veronica arvensis* L.	AZE-IJ-143	Bulaqotu	To increase urination	Genitourinary system conditions	27	93.1	29	Herb	Decoction	Oral	260		0.11	33	0.13
			For swollen veins in the legs	Cardiovascular system conditions	6	20.69									
*Veronica crista-galli* Steven	AZE-IJ-144	Bulaqotu	To increase urination	Genitourinary system conditions	7	26.92	26	Herb	Decoction	Oral	260		0.1	31	0.12
			To improve appetite	Digestive system conditions	15	57.69									
			For swollen veins in the legs	Cardiovascular system conditions	9	34.62									
**Family Polygalaceae**															
*Polygala anatolica* Boiss. & Heldr.	AZE-IJ-145	Südotu	For cough	Respiratory system conditions	22	100	22	Herb	Decoction	Oral	260	0.1	0.08	22	0.08
*Polygala hohenackeriana* Fisch & C.A.Mey.	AZE-IJ-146	Südotu	To help clear phlegm	Respiratory system conditions	30	100	30	Root	Cooking/boiling	Oral	260		0.12	30	0.12
**Family Polygonaceae**															
*Persicaria hydropiper* (L.) Delarbre	AZE-IJ-147	Su bibəri	For women’s health problems	Genitourinary system conditions	10	41.67	24	Herb	Decoction	Oral	260	0.17	0.09	29	0.11
			For stomach ulcers	Digestive system conditions	19	79.17									
*Polygonum aviculare* L.	AZE-IJ-148	Quşbuğdası	To help pass kidney stones	Genitourinary system conditions	40	100	40	Herb	Decoction	Oral	260		0.15	40	0.15
*Rumex tuberosus* L.	AZE-IJ-149	Əvəlik **Murs-ar^L^; Eveluk-ar^L^** **Evelug^Y^** **Fitolli^T^; Futolxə^T^; Tahalu^T^; Turşanç^T^; Xtalo^T^** **Kusuxulaga^X^; Turşag^X^** **Kuzugulag^G^** **Haletin^B^; Mai^B^** **Lurs^G^**	For constipation	Digestive system conditions	51	82.26	62	Root	Cooking/boiling	Oral	260		0.24	67	0.26
			For joint and rheumatic pain	Musculoskeletal conditions	16	25.81									
**Family Portulacaceae**															
*Portulaca oleracea* L.	AZE-IJ-150	Pərpərən **Poy morčigəi^Y^; Muruli^Y^**	For stomach ulcers	Digestive system conditions	27	100	27	Herb	Decoction	Oral	260	0.1	0.1	27	0.1
**Family Primulaceae**															
*Primula ruprechtii* Kusn.	AZE-IJ-151	Novruzçiçəyi **Ğverğver^L^**	For the common cold	Respiratory system conditions	14	51.85	27	Herb	Inhalation	Inhalation	260	0.13	0.1	34	0.13
			For mouth sores	Oral and dental conditions	20	74.07									
**Family Ranunculaceae**															
*Nigella orientalis* L.	AZE-IJ-152	Çörəkotu	For stomach ulcers	Digestive system conditions	34	100	34	Seed	Cooking/boiling	Oral	260	0.09	0.13	34	0.13
*Pulsatilla albana* (Steven) Bercht. & J.Presl	AZE-IJ-153	Güləbətin **Suvantsük^L^; Kambartsük^L^; K’unt’atsük^L^**	For skin problems	Dermatological conditions	24	100	24	Herb	Aqueous/alcoholic extract	Topical	260		0.09	24	0.09
*Thalictrum foetidum* L.	AZE-IJ-154	Qaraqaytaran	For skin problems	Dermatological conditions	16	100	16	Herb	Massage	Topical	260		0.06	16	0.06
**Family Rhamnaceae**															
*Frangula alnus Mill.*	AZE-IJ-155	Mürdəşər	For constipation	Digestive system conditions	38	100	38	Bark	Cooking/boiling	Oral	260	0.1	0.15	38	0.15
*Rhamnus cathartica* L.	AZE-IJ-156	Murdarça	For constipation	Digestive system conditions	16	100	16	Fruit	Cooking/boiling	Oral	260		0.06	16	0.06
**Family Rosaceae**															
*Agrimonia eupatoria* L.	AZE-IJ-157	Kücotu	For stomach and intestinal problems	Digestive system conditions	27	100	27	Herb	Decoction	Oral	260	0.2	0.1	27	0.1
*Alchemilla caucasica* Buser	AZE-IJ-158	Şaxduran	For inflamed gums	Oral and dental conditions	23	71.88	32	Leaf	Decoction	Oral	260		0.12	38	0.15
			For women’s health problems	Genitourinary system conditions	15	46.88									
*Cotoneaster meyeri* Pojark.	AZE-IJ-159	Dovşanalması **Çahabisim^T^**	For constipation	Digestive system conditions	8	100	8	Fruit	Decoction	Oral	260		0.03	8	0.03
*Crataegus caucasica* K.Koch.	AZE-IJ-160	Yemişan **Yaru ini^L^** **Yemşan^T^; Zerü^T^**	To cleanse the blood vessels	Cardiovascular system conditions	22	47.83	46	Fruit	Cooking/boiling	Oral	260		0.18	53	0.2
			For high blood pressure	Cardiovascular system conditions	31	67.39									
*Crataegus pentagyna* Waldst. & Kit. ex Willd.	AZE-IJ-161	Yemişan **Yaru ini^L^** **Yemşan^T^; Zerü^T^**	For high blood pressure	Cardiovascular system conditions	37	75.51	49	Fruit	Cooking/boiling	Oral	260		0.19	56	0.22
			For difficulty sleeping	Nervous system and sleep-related conditions	19	38.78									
*Cydonia oblonga* Mill.	AZE-IJ-162	Heyva **Jum^L^** **Bih^T^** **Cum^G^**	For liver problems	Hepatobiliary conditions	33	50	66	Fruit	Added directly to food	Oral	260		0.25	79	0.3
			To promote bile flow	Hepatobiliary conditions	26	39.39									
			To help clear phlegm	Respiratory system conditions	20	30.3									
*Filipendula ulmaria* (L.) Maxim.	AZE-IJ-163	Quşqonmaz **Karnal^T^; Şabil^T^** **Kində^X^** **Halatai^G^**	For skin problems	Dermatological conditions	18	81.82	22	Herb	Decoction	Oral	260		0.08	25	0.1
			For breathing difficulties	Respiratory system conditions	7	31.82									
*Fragaria vesca* L.	AZE-IJ-164	Çiyələk **T’ap’as-ar^L^; Tamun neq’i-yar^L^**	For the common cold	Respiratory system conditions	44	80	55	Fruit	Used fresh	Oral	260		0.21	60	0.23
			To help pass kidney stones	Genitourinary system conditions	16	29.09									
*Malus orientalis* Uglitzk.	AZE-IJ-165	Şərq alması **K’iri-yar^L^; Ç’uru iç^L^** **Almu^T^; Arma^T^; Cannatalataze^T^; Sif^T^** **Çirməç^X^; Misəmoç^X^** **Çirieç^G^** **Çirieç^B^** **Yeç^G^**	For constipation	Digestive system conditions	16	50	32	Fruit	Decoction	Oral	260		0.12	51	0.2
			For pain and swelling	Respiratory system conditions	23	71.88									
			For headache	Nervous system and sleep-related conditions	12	37.5									
*Potentilla argentea* L.	AZE-IJ-166	Qaytarma **Qazrapats^L^; Tapan neq’i-yar^L^**	For stomach and intestinal problems	Digestive system conditions	15	46.88	32	Rhizome	Cooking/boiling	Oral	260		0.12	41	0.16
			For healing wounds	Dermatological conditions	26	81.25									
*Prunus dulcis* (Mill.) D.A.Webb	AZE-IJ-167	Badam **Badam^L^**	For heart and circulatory problems	Cardiovascular system conditions	46	86.79	53	Fruit	Added directly to food	Oral	260		0.2	59	0.23
			For skin problems	Dermatological conditions	13	24.53									
*Prunus armeniaca* L.	AZE-IJ-168	Ərik **Maşmaş^L^; Qensi^L^** **Qaysi^L^**	For heart and circulatory problems	Cardiovascular system conditions	50	86.21	58	Fruit	Added directly to food	Oral	260		0.22	67	0.26
			For constipation	Digestive system conditions	17	29.31									
*Prunus padus* L.	AZE-IJ-169	Meşə gilası **Martsar^L^; Kits’in ts’ipits’ar^L^**	For stomach and intestinal problems	Digestive system conditions	21	100	21	Fruit	Decoction	Oral	260		0.08	21	0.08
*Pyrus communis* subsp. *caucasica* (Fed.) *Browicz*	AZE-IJ-170	Armud **Qarbi^L^** **Karibə^T^** **Çirçeher^B^** **Ceuhur^G^** **Cühür^G^**	For constipation	Digestive system conditions	17	100	17	Fruit	Added directly to food	Oral	260		0.07	17	0.07
*Rosa canina* L.	AZE-IJ-171	İtburnu **Jik’i-yar^L^** **Çengil^T^** **Çimçe^G^; Çimke^G^; Çimtik^G^; Çunu^G^** **Çunu^B^** **Zinzakay^G^**	For kidney problems	Genitourinary system conditions	39	54.17	72	Fruit	Decoction	Oral	260		0.28	86	0.33
			For liver problems	Hepatobiliary conditions	47	65.28									
*Rosa tomentosa* Sm.	AZE-IJ-172	Tüklü itburnu, həmərsin **Jik’i-yar^L^**	For liver problems	Hepatobiliary conditions	34	35.05	97	Fruit	Decoction	Oral	260		0.37	141	0.54
			For the common cold	Respiratory system conditions	56	57.73									
			For kidney problems	Genitourinary system conditions	51	52.58									
*Rubus ibericus* Juz.	AZE-IJ-173	Böyürtkən **Mere^L^; Fereful^L^** **Pəğala^T^** **Bugürtkan^X^**	For mouth sores	Oral and dental conditions	25	39.06	64	Fruit	Inhalation	Inhalation	260		0.25	79	0.3
			For the common cold	Respiratory system conditions	54	84.38									
*Sorbus aucuparia* L.	AZE-IJ-174	Quşarmudu **Pungul^T^**	For constipation	Digestive system conditions	14	100	14	Fruit	Decoction	Oral	260		0.05	14	0.05
**Family Rubiaceae**															
*Jubogalium setaceum* (Lam.) Kadereit & Schneew.	AZE-IJ-175	Dilqanadan	For pain and swelling	Respiratory system conditions	9	100	9	Herb	Decoction	Oral	260	0.13	0.03	9	0.03
*Rubia tinctorum* L.	AZE-IJ-176	Boyaqotu **Ruğun-ar^L^**	For kidney problems	Genitourinary system conditions	25	53.19	47	Rhizome	Cooking/boiling	Oral	260		0.18	56	0.22
			To increase urination	Genitourinary system conditions	11	23.4									
			To help pass kidney stones	Genitourinary system conditions	20	42.55									
**Family Rutaceae**															
*Dictamnus albus* L.	AZE-IJ-177	Alışan	For skin problems	Dermatological conditions	21	100	21	Herb	Decoction	Oral	260	0.08	0.08	21	0.08
**Family Salicaceae**															
*Salix alba* L.	AZE-IJ-178	Söyüd **Ts’velintar^L^**	For pain and swelling	Respiratory system conditions	27	84.38	32	Bud	Cooking/boiling	Oral	260	0.16	0.12	42	0.16
			For fever	Respiratory system conditions	15	46.88									
**Family Santalaceae**															
*Viscum album* L.	AZE-IJ-179	Öskəotu **Qalçamag^L^; Elenz^L^**	For high blood pressure	Cardiovascular system conditions	13	100	13	Leaf	Decoction	Oral	260	0.05	0.05	13	0.05
**Family Sapindaceae**															
*Acer platanoides* L.	AZE-IJ-180	Ağcaqayın	For pain and swelling	Respiratory system conditions	22	38.6	57	Leaf	Decoction	Oral	260	0.3	0.22	77	0.3
			For fever	Respiratory system conditions	15	26.32									
			For inflamed gums	Oral and dental conditions	40	70.18									
**Family Scrophulariaceae**															
*Scrophularia versicolor* Boiss.	AZE-IJ-181	Qaraşəngi	For pain and swelling	Respiratory system conditions	9	100	9	Herb	Decoction	Oral	260	0.12	0.03	9	0.03
*Verbascum thapsus* L.	AZE-IJ-182	Sığırquyruğu, keçiqulağı **Qhipiq’-ar^L^**	For breathing difficulties	Respiratory system conditions	20	47.62	42	Flower	Decoction	Oral	260		0.16	52	0.2
			For toothache	Oral and dental conditions	14	33.33									
			For irregular heartbeat	Cardiovascular system conditions	18	42.86									
**Family Solanaceae**															
*Alkekengi officinarum* Moench	AZE-IJ-183	Yergiləsi	To increase urination	Genitourinary system conditions	12	100	12	Fruit	Decoction	Oral	260	0.07	0.05	12	0.05
*Atropa bella-donna* L.	AZE-IJ-184	Xanımotu	For joint and rheumatic pain	Musculoskeletal conditions	14	100	14	Leaf	Decoction	Topical	260		0.05	14	0.05
*Hyoscyamus niger* L.	AZE-IJ-185	Batbat	For joint and rheumatic pain	Musculoskeletal conditions	26	100	26	Leaf	Decoction	Topical	260		0.1	26	0.1
**Family Taxaceae**															
*Taxus baccata* L.	AZE-IJ-186	Qaraçöhrə	For skin problems	Dermatological conditions	23	100	23	Leaf	Decoction	Topical	260	0.09	0.09	23	0.09
**Family Thymelaeaceae**															
*Daphne mezereum* L.	AZE-IJ-187	Canavargiləsi	For joint and rheumatic pain	Musculoskeletal conditions	17	100	17	Leaf	Decoction	Oral	260	0.07	0.07	17	0.07
**Family Verbenaceae**															
*Verbena officinalis* L.	AZE-IJ-188	Minaçiçəyi	To help pass kidney stones	Genitourinary system conditions	19	82.61	23	Herb	Fruit juice	Oral	260	0.12	0.09	31	0.12
			For women’s health problems	Genitourinary system conditions	12	52.17									
**Family Viburnaceae**															
*Sambucus nigra* L.	AZE-IJ-189	Gəndəlaş **Serk’v^L^; Kirf^L^; Qerf^L^**	For the common cold	Respiratory system conditions	40	78.43	51	Flower	Decoction	Oral	260	0.15	0.2	57	0.22
			For joint and rheumatic pain	Musculoskeletal conditions	17	33.33									
*Viburnum lantana* L.	AZE-IJ-190	Başınağacı **Fart-ar^L^**	For women’s health problems	Genitourinary system conditions	22	100	22	Fruit	Decoction	Oral	260		0.08	22	0.08
**Family Violaceae**															
*Viola caucasica* Kolenati	AZE-IJ-191	Bənövşə **Banofşa^T^**	For fever	Respiratory system conditions	27	90	30	Leaf	Decoction	Oral	260	0.15	0.12	41	0.16
			To increase urination	Genitourinary system conditions	14	46.67									
*Viola rupestris* F.W.Schmidt.	AZE-IJ-192	Bənövşə **Banofşa^T^**	To help clear phlegm	Respiratory system conditions	36	100	36	Herb	Decoction	Oral	260		0.14	36	0.14
**Family Urticaceae**															
*Urtica dioica* L.	AZE-IJ-193	Gicitkən **Werg^L^** **Mɨčʼ^X^** **Meǯ^G^** **Meǯ^B^; ʕangut^B^** **Gezine^Y^** **Cezina^T^; Gicitkan^T^** **Meç^X^; Mişə^X^** **Meç^G^**, **Meç^B^**, **Mec^G^**	For women’s health problems	Genitourinary system conditions	23	40.35	57	Leaf	Decoction	Oral	260	0.25	0.22	66	0.25
			For stomach ulcers	Digestive system conditions	16	28.07									
			For kidney problems	Genitourinary system conditions	27	47.37									

**Table 5:** Rare and locality-specific ethnomedicinal reports excluded from the principal quantitative analyses.

Settlement	Scientific Name and Voucher Code	Voucher Code	FC	Plant Part Used	Reported Use
Khinalig	*Rumex alpinus* L.	AZE-IJ-194	1	Root	Constipation
Khinalig	*Dracocephalum austriacum* L.	AZE-IJ-195	2	Aerial parts	Gastrointestinal disorders
Khinalig	*Betonica nivea* Steven	AZE-IJ-196	3	Aerial parts	Respiratory conditions
Budug	*Sonchus oleraceus* L.	AZE-IJ-197	1	Aerial parts	Kidney disorders
Haputlu	*Salvia sclarea* L.	AZE-IJ-198	3	Leaf	Common cold
Adur	*Thymus daghestanicus* Klokov & Des.-Shost.	AZE-IJ-199	2	Aerial parts	Respiratory conditions
Kryz	*Prunella laciniata* (L.) L.	AZE-IJ-200	3	Aerial parts	Gastrointestinal disorders
Kryz	*Taraxacum syriacum* Boiss.	AZE-IJ-201	1	Root	Atherosclerosis
Afurja	*Juniperus excelsa subsp. polycarpos* (K.Koch) Takht.	AZE-IJ-202	2	Ripe fruits	Diuretic
Gechresh	*Achillea filipendulina Lam.*	AZE-IJ-203	4	Aerial parts	Hemorrhoids; prepared as an infusion
Galaykhudat	*Tanacetum punctatum* (Desr.) Grierson	AZE-IJ-204	3	Flowers	Diarrhea

**Table 6:** Informant consensus factor for broad health-condition categories based on 8682 use-reports.

Broad Health-Condition Category	Nt	Nur	ICF
Cardiovascular system conditions	29	855	0.967
Dermatological conditions	46	1123	0.960
Digestive system conditions	53	1422	0.963
Endocrine and metabolic conditions	11	261	0.962
Genitourinary system conditions	43	1138	0.963
Hepatobiliary conditions	17	546	0.971
Musculoskeletal conditions	10	166	0.945
Nervous system and sleep-related conditions	21	661	0.970
Oral and dental conditions	19	453	0.960
Respiratory system conditions	64	2057	0.969

**Note:** Nt represents the number of medicinal plant taxa cited within each broad health-condition category, and Nur represents the number of use-reports. ICF was calculated as (Nur − Nt)/(Nur − 1). Local use descriptions were recorded first and retained in the principal taxa table; the broad categories shown here were used only for secondary analytical organization.

**Table 7:** Additional ethnobotanical uses of the medicinal plants documented in the Guba District.

Use Category	No. of Species	Plant Material or Mode of Use	Local Application
Food plants	35	Various edible plant parts	Eaten raw or added to cooked dishes
Spices and flavouring agents	12	Mainly aromatic aerial parts, fruits, and seeds	Used to flavour foods
Fodder plants	8	Aerial parts	Used as livestock feed
Natural dye sources	10	Fruits, flowers, bulb skins, bark, fruit peels, roots, and rhizomes	Used to dye wool and traditional textiles
Fuelwood	11	Stems, branches, and other woody parts	Traditionally used as fuel; use has declined
Construction materials	6	Mainly trunks and other woody parts	Used for construction, furniture, and household objects
Melliferous plants	22	Flowering parts	Recognized as honeybee forage
Ornamental plants	12	Whole plants	Grown in gardens and along roadsides

**Note:** Individual species could be assigned to more than one use category; therefore, the category totals are not additive. Detailed species-level data are provided in Table S2.

**Table 8:** Medicinal plant species included in the national conservation lists of Azerbaijan.

Conservation List	Family	Species
Red Book	Amaryllidaceae	*Allium oreophilum* C.A.Mey.
	Apiaceae	*Bupleurum wittmannii* Steven
		*Carum caucasicum* (M.Bieb.) Boiss.
		*Ferula caspica* M.Bieb.
	Asphodelaceae	*Eremurus azerbajdzhanicus* Kharkev.
	Asteraceae	*Tanacetum coccineum* (Willd.) Grierson
	Fagaceae	*Quercus castaneifolia* C.A.Mey.
	Orchidaceae	*Ophrys apifera* Huds.
		*Orchis mascula* (L.) L.
	Primulaceae	*Primula ruprechtii* Kusn.
	Rubiaceae	*Jubogalium setaceum* (Lam.) Kadereit & Schneew.
	Taxaceae	*Taxus baccata* L.
	Lythraceae	*Punica granatum* L.
	Violaceae	*Viola rupestris* F.W.Schmidt.
Pink List	Amaryllidaceae	*Allium ursinum* L.
		*Galanthus alpinus* Sosn. var. *alpinus*
	Anacardiaceae	*Rhus coriaria* L.
	Campanulaceae	*Asyneuma campanuloides* (M.Bieb.) Bornm.
	Colchicaceae	*Colchicum speciosum* Steven
	Malvaceae	*Alcea kusjariensis* (Iljin ex Grossh.) Iljin
	Ranunculaceae	*Pulsatilla albana* (Steven) Bercht. & J. Presl.
	Rosaceae	*Sorbus aucuparia* L.
	Solanaceae	*Atropa bella-donna* L.
	Violaceae	*Viola caucasica* Kolenati.

**Table 9:** Comparison of medicinal plant uses recorded in the present study with selected primary ethnobotanical studies from Azerbaijan and neighboring Caucasian regions.

Taxon	Reported Use in the Present Study	Plant Part; Preparation; Route	Previous Report from Azerbaijan	Previous Report from Neighboring Caucasian Regions	Comparative Status and Supporting References
*Allium oreophilum* C.A.Mey.	For the common cold	Bulb; Used fresh; Oral	Same species–use combination not identified in the selected Azerbaijan sources.	Same species–use combination not identified in the selected Georgian, Armenian, and Dagestani sources.	Potentially novel within the consulted comparison corpus [11,12,14,15,26,27,28,29,30,33,34].
*Ferula caspica* M.Bieb.	For skin problems	Root; Cooking/boiling; Oral	Same species–use combination not identified in the selected Azerbaijan sources.	Same species–use combination not identified in the selected Georgian, Armenian, and Dagestani sources.	Potentially novel within the consulted comparison corpus [11,12,14,15,26,27,28,29,30,33,34].
*Heracleum sosnowskyi* Manden.	For skin problems	Fruit; Fruit juice; Oral	Same species–use combination not identified in the selected Azerbaijan sources.	Same species–use combination not identified in the selected Georgian, Armenian, and Dagestani sources.	Potentially novel within the consulted comparison corpus [11,12,14,15,26,27,28,29,30,33,34].
*Anthemis cotula* L.	For headache	Flower; Decoction; Oral	Same species–use combination not identified in the selected Azerbaijan sources.	Same species–use combination not identified in the selected Georgian, Armenian, and Dagestani sources.	Potentially novel within the consulted comparison corpus [11,12,14,15,26,27,28,29,30,33,34].
*Centaurea phrygia* subsp. *salicifolia* (M.Bieb. ex Willd.) Mikheev	For fever	Flower; Decoction; Oral	Same species–use combination not identified in the selected Azerbaijan sources.	Same species–use combination not identified in the selected Georgian, Armenian, and Dagestani sources.	Potentially novel within the consulted comparison corpus [11,12,14,15,26,27,28,29,30,33,34].
*Lactuca tatarica* (L.) C.A.Mey.	To improve appetite	Leaf; Added directly to food; Oral	Same species–use combination not identified in the selected Azerbaijan sources.	Same species–use combination not identified in the selected Georgian, Armenian, and Dagestani sources.	Potentially novel within the consulted comparison corpus [11,12,14,15,26,27,28,29,30,33,34].
*Pseudopodospermum szowitzii* (DC.) Kuth.	For skin problems	Root; Cooking/boiling; Oral	Same species–use combination not identified in the selected Azerbaijan sources.	Same species–use combination not identified in the selected Georgian, Armenian, and Dagestani sources.	Potentially novel within the consulted comparison corpus [11,12,14,15,26,27,28,29,30,33,34].
*Tanacetum coccineum* (Willd.) Grierson	For stomach and intestinal problems	Flower; Decoction; Oral	Same species–use combination not identified in the selected Azerbaijan sources.	Same species–use combination not identified in the selected Georgian, Armenian, and Dagestani sources.	Potentially novel within the consulted comparison corpus [11,12,14,15,26,27,28,29,30,33,34].
*Taraxacum sonchoides* (D.Don) Sch.Bip.	To cleanse the blood vessels	Root; Cooking/boiling; Oral	Same species–use combination not identified in the selected Azerbaijan sources.	Same species–use combination not identified in the selected Georgian, Armenian, and Dagestani sources.	Potentially novel within the consulted comparison corpus [11,12,14,15,26,27,28,29,30,33,34].
*Tragopogon tuberosus* K.Koch.	For high blood pressure	Leaf; Decoction; Oral	Same species–use combination not identified in the selected Azerbaijan sources.	Same species–use combination not identified in the selected Georgian, Armenian, and Dagestani sources.	Potentially novel within the consulted comparison corpus [11,12,14,15,26,27,28,29,30,33,34].
*Anchusa azurea* Mill.	For women’s health problems	Herb; Decoction; Oral	Same species–use combination not identified in the selected Azerbaijan sources.	Same species–use combination not identified in the selected Georgian, Armenian, and Dagestani sources.	Potentially novel within the consulted comparison corpus [11,12,14,15,26,27,28,29,30,33,34].
*Asyneuma campanuloides* (M.Bieb.) Bornm.	For joint and rheumatic pain	Herb; Decoction; Oral	Same species–use combination not identified in the selected Azerbaijan sources.	Same species–use combination not identified in the selected Georgian, Armenian, and Dagestani sources.	Potentially novel within the consulted comparison corpus [11,12,14,15,26,27,28,29,30,33,34].
*Campanula glomerata* L.	For pain and swelling	Herb; Aqueous/alcoholic extract; Oral	Same species–use combination not identified in the selected Azerbaijan sources.	Same species–use combination not identified in the selected Georgian, Armenian, and Dagestani sources.	Potentially novel within the consulted comparison corpus [11,12,14,15,26,27,28,29,30,33,34].
*Astragalus caucasicus* Pall.	For skin problems	Herb; Fruit juice; Oral	Same species–use combination not identified in the selected Azerbaijan sources.	Same species–use combination not identified in the selected Georgian, Armenian, and Dagestani sources.	Potentially novel within the consulted comparison corpus [11,12,14,15,26,27,28,29,30,33,34].
*Medicago* × varia Martyn	For skin problems	Herb; Poultice; Topical	Same species–use combination not identified in the selected Azerbaijan sources.	Same species–use combination not identified in the selected Georgian, Armenian, and Dagestani sources.	Potentially novel within the consulted comparison corpus [11,12,14,15,26,27,28,29,30,33,34].
*Medicago minima* (L.) Bartal.	For healing wounds	Herb; Decoction; Oral	Same species–use combination not identified in the selected Azerbaijan sources.	Same species–use combination not identified in the selected Georgian, Armenian, and Dagestani sources.	Potentially novel within the consulted comparison corpus [11,12,14,15,26,27,28,29,30,33,34].
*Onobrychis petraea* (M.Bieb. ex Willd.) Fisch.	For diabetes	Herb; Decoction; Oral	Same species–use combination not identified in the selected Azerbaijan sources.	Same species–use combination not identified in the selected Georgian, Armenian, and Dagestani sources.	Potentially novel within the consulted comparison corpus [11,12,14,15,26,27,28,29,30,33,34].
*Onobrychis radiata* (Desf.) M.Bieb	For pain and swelling	Herb; Decoction; Oral	Same species–use combination not identified in the selected Azerbaijan sources.	Same species–use combination not identified in the selected Georgian, Armenian, and Dagestani sources.	Potentially novel within the consulted comparison corpus [11,12,14,15,26,27,28,29,30,33,34].
*Trifolium ochroleucon* subsp. *ochroleucon*	For pain and swelling	Herb; Decoction; Oral	Same species–use combination not identified in the selected Azerbaijan sources.	Same species–use combination not identified in the selected Georgian, Armenian, and Dagestani sources.	Potentially novel within the consulted comparison corpus [11,12,14,15,26,27,28,29,30,33,34].
*Hypericum linarioides* Bosse	For difficulty sleeping	Herb; Decoction; Oral	Same species–use combination not identified in the selected Azerbaijan sources.	Same species–use combination not identified in the selected Georgian, Armenian, and Dagestani sources.	Potentially novel within the consulted comparison corpus [11,12,14,15,26,27,28,29,30,33,34].
*Geranium columbinum* L.	For mouth sores	Herb; Inhalation; Inhalation	Same species–use combination not identified in the selected Azerbaijan sources.	Same species–use combination not identified in the selected Georgian, Armenian, and Dagestani sources.	Potentially novel within the consulted comparison corpus [11,12,14,15,26,27,28,29,30,33,34].
*Dracocephalum botryoides* Steven	For kidney problems	Herb; Decoction; Oral	Same species–use combination not identified in the selected Azerbaijan sources.	Same species–use combination not identified in the selected Georgian, Armenian, and Dagestani sources.	Potentially novel within the consulted comparison corpus [11,12,14,15,26,27,28,29,30,33,34].
*Marrubium vulgare* L.	For skin problems	Herb; Aqueous/alcoholic extract; Topical	Same species–use combination not identified in the selected Azerbaijan sources.	Same species–use combination not identified in the selected Georgian, Armenian, and Dagestani sources.	Potentially novel within the consulted comparison corpus [11,12,14,15,26,27,28,29,30,33,34].
*Nepeta betonicifolia* C.A.Mey.	For women’s health problems	Leaf; Decoction; Oral	Same species–use combination not identified in the selected Azerbaijan sources.	Same species–use combination not identified in the selected Georgian, Armenian, and Dagestani sources.	Potentially novel within the consulted comparison corpus [11,12,14,15,26,27,28,29,30,33,34].
*Prunella vulgaris* L.	For pain and swelling	Herb; Decoction; Oral	Same species–use combination not identified in the selected Azerbaijan sources.	Same species–use combination not identified in the selected Georgian, Armenian, and Dagestani sources.	Potentially novel within the consulted comparison corpus [11,12,14,15,26,27,28,29,30,33,34].
*Sideritis montana* L.	For pain and swelling	Root; Cooking/boiling; Oral	Same species–use combination not identified in the selected Azerbaijan sources.	Same species–use combination not identified in the selected Georgian, Armenian, and Dagestani sources.	Potentially novel within the consulted comparison corpus [11,12,14,15,26,27,28,29,30,33,34].
*Stachys pauli* Grossh.	For fever	Herb; Decoction; Oral	Same species–use combination not identified in the selected Azerbaijan sources.	Same species–use combination not identified in the selected Georgian, Armenian, and Dagestani sources.	Potentially novel within the consulted comparison corpus [11,12,14,15,26,27,28,29,30,33,34].
*Linum austriacum* L.	For healing wounds	Fruit; Decoction; Oral	Same species–use combination not identified in the selected Azerbaijan sources.	Same species–use combination not identified in the selected Georgian, Armenian, and Dagestani sources.	Potentially novel within the consulted comparison corpus [11,12,14,15,26,27,28,29,30,33,34].
*Alcea kusjariensis* (Iljin ex Grossh.) Iljin	For breathing difficulties	Leaf; Decoction; Oral	Same species–use combination not identified in the selected Azerbaijan sources.	Same species–use combination not identified in the selected Georgian, Armenian, and Dagestani sources.	Potentially novel within the consulted comparison corpus [11,12,14,15,26,27,28,29,30,33,34].
*Chrysojasminum fruticans* (L.) Banfi	For healing wounds	Leaf; Fruit juice; Oral	Same species–use combination not identified in the selected Azerbaijan sources.	Same species–use combination not identified in the selected Georgian, Armenian, and Dagestani sources.	Potentially novel within the consulted comparison corpus [11,12,14,15,26,27,28,29,30,33,34].
*Jubogalium setaceum* (Lam.) Kadereit & Schneew.	For pain and swelling	Herb; Decoction; Oral	Same species–use combination not identified in the selected Azerbaijan sources.	Same species–use combination not identified in the selected Georgian, Armenian, and Dagestani sources.	Potentially novel within the consulted comparison corpus [11,12,14,15,26,27,28,29,30,33,34].
*Viola rupestris* F.W.Schmidt.	To help clear phlegm	Herb; Decoction; Oral	Same species–use combination not identified in the selected Azerbaijan sources.	Same species–use combination not identified in the selected Georgian, Armenian, and Dagestani sources.	Potentially novel within the consulted comparison corpus [11,12,14,15,26,27,28,29,30,33,34].
*Carpinus orientalis* Mill.	For skin problems	Leaf; Poultice; Topical	Same species–use combination not identified in the selected Azerbaijan sources.	Same species–use combination not identified in the selected Georgian, Armenian, and Dagestani sources.	Potentially novel within the consulted comparison corpus [11,12,14,15,26,27,28,29,30,33,34].
*Dipsacus laciniatus* L.	For women’s health problems	Flower; Decoction; Oral	Same species–use combination not identified in the selected Azerbaijan sources.	Same species–use combination not identified in the selected Georgian, Armenian, and Dagestani sources.	Potentially novel within the consulted comparison corpus [11,12,14,15,26,27,28,29,30,33,34].
*Lomelosia caucasica* (M.Bieb.) Greuter & Burdet	For diabetes	Flower; Decoction; Oral	Same species–use combination not identified in the selected Azerbaijan sources.	Same species–use combination not identified in the selected Georgian, Armenian, and Dagestani sources.	Potentially novel within the consulted comparison corpus [11,12,14,15,26,27,28,29,30,33,34].
*Colchicum trigynum* (Steven ex Adams) Stearn	For pain and swelling	Tuber; Decoction; Oral	Same species–use combination not identified in the selected Azerbaijan sources.	Same species–use combination not identified in the selected Georgian, Armenian, and Dagestani sources.	Potentially novel within the consulted comparison corpus [11,12,14,15,26,27,28,29,30,33,34].

**Note:** The comparison was conducted at the species–use level. “Not identified” means that the same species–use combination was not found in the selected sources consulted for this study; it does not establish global novelty. Accordingly, these records are described as potentially novel within the defined comparison corpus. Sources: Bussmann et al. 2016, 2018 [11,12]; Pieroni and Sõukand 2019 [14]; Sõukand and Pieroni 2019 [15]; Aliev et al. 2023 [26]; Kazancı et al. 2020 [27]; Nanagulyan et al. 2020 [28]; Hovsepyan et al. 2019 [29]; Pieroni et al. 2021 [30]; Isayev et al. 2026 [33,34].

## Data Availability

The data presented in this study are available in the article and its Supplementary Materials. Participant-level interview data and the species × informant matrix are not publicly available because of confidentiality considerations and the potential risk of indirect re-identification within small ethnolinguistic communities. No standalone analysis script was generated for this study.

## Supplementary Materials

The following supporting information can be downloaded at: https://www.mdpi.com/article/10.3390/plants15193009/s1, Table S1: Number of documented medicinal plant species and Family Use Value (FUV) by botanical family in the Guba District of Azerbaijan; Table S2: Additional Ethnobotanical Uses of Medicinal Plants Documented in the Guba District.

## Abbreviations

The following abbreviations are used in this manuscript:

FC	Frequency of Citation
FL	Fidelity Level
FUV	Family Use Value
ICD-11	International Classification of Diseases 11th Revision
ICF	Informant Consensus Factor
ISE	International Society of Ethnobiology
POWO	Plants of the World Online
RFC	Relative Frequency of Citation
UV	Use Value
