# Supplementary material for: Traditional Medicinal Plant Knowledge in the Guba District of Azerbaijan: An Ethnobotanical Study
Source: Plants (Basel). 2026 Sep 30;15(19):3009. doi: 10.3390/plants15193009 (PMC13666791; doi:10.3390/plants15193009)
Supplement: Supplementary file 1 [file plants-15-03009-s001.zip › plants-4567296-supplementary.pdf]

**Table S1.** Number of documented medicinal plant species and Family Use Value (FUV) by botanical family in the Guba District of Azerbaijan.

| Plant Family | Number of Plant Species | FUV |
|---|---|---|
| Amaranthaceae | 1 | 0.28 |
| Amaryllidaceae | 5 | 0.16 |
| Anacardiaceae | 2 | 0.23 |
| Apiaceae | 10 | 0.18 |
| Asphodelaceae | 1 | 0.15 |
| Asteraceae | 19 | 0.21 |
| Berberidaceae | 1 | 0.18 |
| Betulaceae | 5 | 0.13 |
| Boraginaceae | 2 | 0.09 |
| Brassicaceae | 4 | 0.22 |
| Campanulaceae | 3 | 0.08 |
| Cannabaceae | 1 | 0.22 |
| Capparaceae | 1 | 0.13 |
| Caprifoliaceae | 6 | 0.15 |
| Chenopodiaceae | 3 | 0.14 |
| Colchicaceae | 2 | 0.06 |
| Cornaceae | 1 | 0.21 |
| Cucurbitaceae | 3 | 0.12 |
| Cupressaceae | 1 | 0.07 |
| Elaeagnaceae | 2 | 0.31 |
| Equisetaceae | 1 | 0.29 |
| Fabaceae | 13 | 0.13 |
| Fagaceae | 2 | 0.15 |
| Hypericaceae | 2 | 0.30 |
| Gentianaceae | 3 | 0.13 |
| Geraniaceae | 2 | 0.18 |
| Juglandaceae | 1 | 0.25 |
| Lamiaceae | 22 | 0.23 |
| Linaceae | 1 | 0.07 |
| Lythraceae | 1 | 0.29 |
| Malvaceae | 5 | 0.22 |
| Moraceae | 2 | 0.29 |
| Nitrariaceae | 1 | 0.16 |
| Oleaceae | 2 | 0.06 |
| Onagraceae | 1 | 0.08 |
| Orchidaceae | 3 | 0.15 |
| Papaveraceae | 3 | 0.17 |
| Pedaliaceae | 1 | 0.23 |

| | | |
|---|---|---|
| Plantaginaceae | 5 | 0.17 |
| Polygalaceae | 2 | 0.10 |
| Polygonaceae | 3 | 0.17 |
| Portulacaceae | 1 | 0.10 |
| Primulaceae | 1 | 0.13 |
| Ranunculaceae | 3 | 0.09 |
| Rhamnaceae | 2 | 0.10 |
| Rosaceae | 18 | 0.20 |
| Rubiaceae | 2 | 0.13 |
| Rutaceae | 1 | 0.08 |
| Salicaceae | 1 | 0.16 |
| Santalaceae | 1 | 0.05 |
| Sapindaceae | 1 | 0.30 |
| Scrophulariaceae | 2 | 0.12 |
| Solanaceae | 3 | 0.07 |
| Taxaceae | 1 | 0.09 |
| Thymelaeaceae | 1 | 0.07 |
| Verbenaceae | 1 | 0.12 |
| Viburnaceae | 2 | 0.15 |
| Violaceae | 2 | 0.15 |
| Urticaceae | 1 | 0.25 |

**Table S2.** Additional Ethnobotanical Uses of Medicinal Plants Documented in the Guba District

| Use category | Category total (n) | Accepted scientific names | Reported use/remarks |
|---|---|---|---|
| Food plants | 35 | *Amaranthus retroflexus* L.; *Beta vulgaris* L.; *Spinacia oleracea* L.; *Allium cepa* L.; *Allium sativum* L.; *Allium ursinum* L.; *Prunus amygdalus* Batsch.; *Prunus armeniaca* L.; *Brassica oleracea* L.; *Capsella bursa-pastoris* Medik.; *Cornus mas* L.; *Corylus avellana* L.; *Cucurbita pepo* L.; *Cydonia oblonga* Mill.; *Elaeagnus angustifolia* L.; *Eremurus azerbajdzhanicus* Kharkev.; *Fagus orientalis* Lipsky; *Juglans regia* L.; *Ficus carica* L.; *Fragaria vesca* L.; *Malva sylvestris* L.; *Malus orientalis* Uglitzk.; *Morus alba* L.; *Prangos ferulacea* (L.) Lindl.; *Portulaca oleracea* L.; *Pyrus communis* subsp. *caucasica* (Fed.) Browicz; *Punica granatum* L.; *Raphanus raphanistrum* subsp. *sativus* (L.) Schmalh.; *Pseudopodospermum leptophyllum* (DC.) Kuth.; *Rubus ibericus* Juz.; *Rumex tuberosus* L.; *Sesamum indicum* L.; *Sorbus aucuparia* L.; *Tragopogon tuberosus* C. Koch.; *Vicia lathyroides* L. | Consumed as food; eaten raw and/or added to cooked dishes. |
| Spices and flavouring agents | 12 | *Anethum graveolens* L.; *Berberis vulgaris* L.; *Carum caucasicum* (Bieb) Boiss.; *Coriandrum sativum* L.; *Foeniculum vulgare* Mill.; *Mentha aquatica* L.; *Mentha longifolia* (L.) Huds.; *Nepeta betonicifolia* C.A.Mey; *Pimpinella anisum* L.; *Rhus coriaria* L.; *Thymus praecox* subsp. *caucasicus* (Willd. ex Ronniger) Jalas; *Ziziphora capitata* L. | Used as a spice or flavouring agent during food preparation. |
| Fodder plants | 8 | *Astragalus caucasicus* Pall.; *Lathyrus hirsutus* L.; *Medicago* × varia Martyn; *Medicago minima* (L.) Bartal.; *Onobrychis petraea* (Bieb. ex Willd.) Fisch.; *Onobrychis radiata* (Desv.) Bieb; *Trigonella spicata* Sibth. et Smith.; *Vicia lathyroides* L. | Aerial parts used as fodder for livestock. |
| Natural dye sources | 10 | *Rhus coriaria* L. (fruits; crimson dye); *Sambucus nigra* L. (fruits; dark-purple dye); *Alcea kusjariensis* (Iljin ex Grossh.) Iljin (petals); *Calendula arvensis* L. (flowers; dark-orange dye); *Helichrysum plinthocalyx* (C.Koch) Sosn. (petals); *Glaucium elegans* Fisch. et C.A.Mey. (petals); *Allium cepa* L. (bulb skins); *Frangula alnus* Mill. (bark of stems and branches); *Punica granatum* L. (fruit peels; dark-brown dye); *Rubia tinctorum* L. (roots and rhizomes) | Used to prepare aqueous natural dyes for wool yarn and traditional textiles. |

| | | | |
|---|---|---|---|
| Fuelwood | 11 | *Acer platanoides* L.; *Alnus glutinosa* subsp. *barbata* (C.A.Mey.) Yalt.; *Alnus incana* (L.) Moench.; *Betula pendula* Roth.; *Carpinus orientalis* Mill.; *Cotinus coggygria* Scop.; *Elaeagnus angustifolia* L.; *Fagus orientalis* Lipsky; *Quercus castaneifolia* C.A.Mey.; *Salix alba* L.; *Tilia cordata* Mill. | Stems, branches, and other woody parts traditionally used as fuelwood; use has declined following regional gasification and conservation measures. |
| Construction materials | 6 | *Alnus glutinosa* subsp. *barbata* (C.A.Mey.) Yalt.; *Alnus incana* (L.) Moench.; *Elaeagnus angustifolia* L.; *Fagus orientalis* Lipsky; *Quercus castaneifolia* C.A.Mey.; *Tilia cordata* Mill. | Trunks and other woody parts used in construction and in the production of furniture and household objects. |
| Melliferous plants | 22 | *Prunus amygdalus* Batsch.; *Prunus armeniaca* L.; *Betonica officinalis* L.; *Centaurea phrygia* subsp. *salicifolia* (M.Bieb. ex Willd.) Mikheev.; *Elaeagnus angustifolia* L.; *Eremurus azerbajdzhanicus* Kharkev.; *Ferula caspica* M. Bieb.; *Glycyrrhiza glabra* L.; *Lamium album* L.; *Medicago* × varia Martyn; *Melissa officinalis* L.; *Nepeta betonicifolia* C.A.Mey; *Onobrychis petraea* (Bieb. ex Willd.) Fisch.; *Onobrychis radiata* (Desv.) Bieb; *Phlomis herba-venti* subsp. *pungens* (Willd.) Maire ex DeFilipps; *Rosa canina* L.; *Salvia aethiopis* L.; *Salvia glutinosa* L.; *Silybum marianum* (L.) Gaertn.; *Teucrium chamaedrys* L.; *Thymus praecox* subsp. *caucasicus* (Willd. ex Ronniger) Jalas; *Zosima absinthiifolia* (Vent.) Link | Recognized by informants as a plant visited by honeybees during flowering. |
| Ornamental plants | 12 | *Eremurus azerbajdzhanicus* Kharkev.; *Galanthus alpinus* var. *alpinus*; *Chrysojasminum fruticans* (L.) Banfi; *Lamium album* L.; *Lonicera iberica* Bieb.; *Melissa officinalis* L.; *Salix alba* L.; *Sorbus aucuparia* L.; *Tilia cordata* Mill.; *Viburnum lantana* L.; *Viola caucasica* Kolenati.; *Viola rupestris* F.W. Schmidt. | Cultivated in home gardens and along roadsides in settlements. |
